# DDR1 autophosphorylation is a result of aggregation into dense clusters

**DOI:** 10.1038/s41598-019-53176-4

**Published:** 2019-11-19

**Authors:** David S. Corcoran, Victoria Juskaite, Yuewei Xu, Frederik Görlitz, Yuriy Alexandrov, Christopher Dunsby, Paul M. W. French, Birgit Leitinger

**Affiliations:** 1 National Heart and Lung Institute, Imperial College London, London, SW7 2AZ UK; 2 Photonics Group, Physics Department, Imperial College London, London, SW7 2AZ UK; 3 Facility for Imaging by Light Microscopy, Imperial College London, London, SW7 2AZ UK; 4 Present Address: Department of Biological Science, Florida State University, Tallahassee, FL 32304 USA; 5 Present Address: LifeArc, Open Innovation Campus, Stevenage, SG1 2FX UK; 6 The Francis Crick Institute, 1 Midland Road, London, UK

**Keywords:** Kinases, Growth factor signalling, Extracellular matrix, Cellular imaging

## Abstract

The collagen receptor DDR1 is a receptor tyrosine kinase that promotes progression of a wide range of human disorders. Little is known about how ligand binding triggers DDR1 kinase activity. We previously reported that collagen induces DDR1 activation through lateral dimer association and phosphorylation between dimers, a process that requires specific transmembrane association. Here we demonstrate ligand-induced DDR1 clustering by widefield and super-resolution imaging and provide evidence for a mechanism whereby DDR1 kinase activity is determined by its molecular density. Ligand binding resulted in initial DDR1 reorganisation into morphologically distinct clusters with unphosphorylated DDR1. Further compaction over time led to clusters with highly aggregated and phosphorylated DDR1. Ligand-induced DDR1 clustering was abolished by transmembrane mutations but did not require kinase activity. Our results significantly advance our understanding of the molecular events underpinning ligand-induced DDR1 kinase activity and provide an explanation for the unusually slow DDR1 activation kinetics.

## Introduction

Receptor tyrosine kinases (RTKs) are key signalling receptors that mediate fundamental cellular responses. The molecular events underpinning the process of ligand-induced kinase activation have been revealed for a number of well-studied RTKs, including epidermal growth factor (EGF) and insulin receptors^1,2^. However, little is known about this process for the discoidin domain receptors, DDR1 and DDR2. The DDRs are collagen receptors whose aberrant functions contribute to disease progression of a wide range of human disorders, including arthritis, fibrosis and many types of cancer^3^. While the binding of the collagen triple helix to the ligand-binding DDR discoidin domain is known at atomic-level detail^4^, mechanistic insight into how ligand binding induces intracellular kinase activation has been lacking. The DDRs form constitutive dimers in the absence of collagen^5–7^, hence the canonical model of ligand-induced RTK dimerisation cannot account for DDR kinase activation. Moreover, conformational changes within dimers were ruled out as a triggering mechanism^7^. Ligand-induced DDR kinase autophosphorylation occurs with unusually slow kinetics^8,9^, a phenomenon that still awaits a mechanistic explanation. We recently reported biochemical evidence for phosphorylation between neighbouring DDR1 dimers^10^, a process that can only occur if DDR1 dimers are closely apposed, most likely in densely packed clusters. We also showed collagen-induced clustering of DDR1 on the surface of cells^10^, in agreement with a previous study on DDR1 tagged with fluorescent proteins^6^. Other studies have also observed collagen-induced clusters of DDR1 in a number of cell types and under various conditions^6,11–13^. However, how clustering of DDR1 leads to autophosphorylation, and whether phosphorylated DDR1 correlates with DDR1 aggregated in dense clusters, was not explored.

In the present study, we used imaging to dissect the process of DDR1 kinase activation into two distinct stages. In the first stage, within 5 minutes of collagen binding, DDR1 redistributes into morphologically distinct clusters that contain unphosphorylated DDR1. In the second stage, DDR1 aggregates further, over the course of 45–60 minutes, and this results in more densely packed structures that contain phosphorylated DDR1. Our data show that clustering requires the DDR1 transmembrane region and suggest a mechanism whereby DDR1 kinase activity is determined by molecular density. Thus, we have found a simple explanation for the unusually slow DDR1 activation kinetics.

## Results

**Figure 1:**
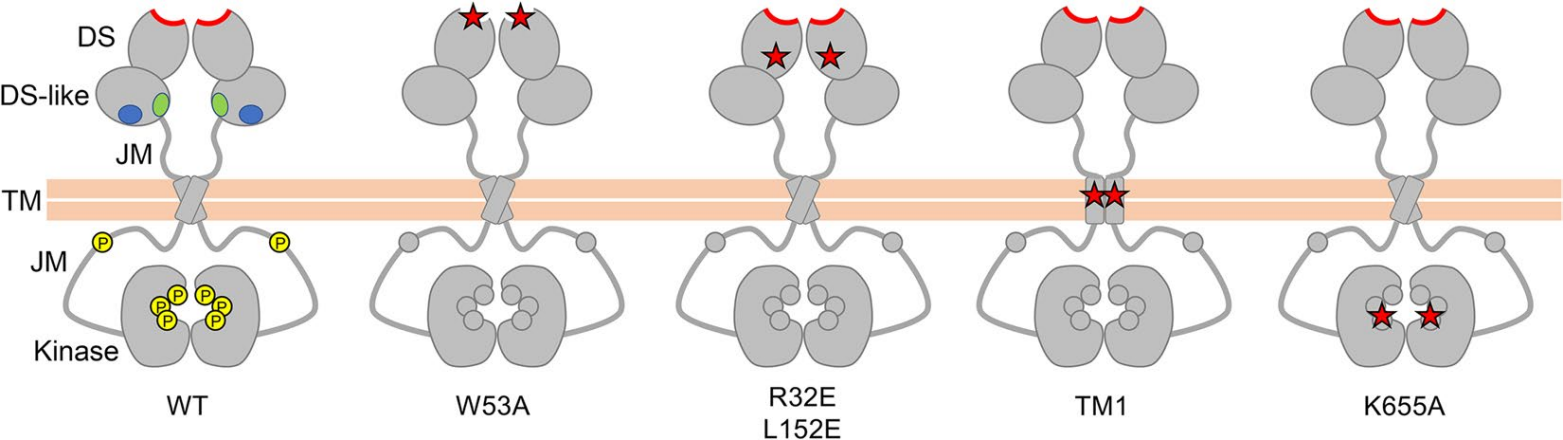
Schematic diagram of wild-type and signalling-defective DDR1 mutants. The extracellular region consists of two globular domains, the N-terminal discoidin (DS) domain and the discoidin-like (DS-like) domain, followed by a highly flexible juxtamembrane (JM) region. The transmembrane (TM) region contains a dimerisation motif. The intracellular catalytic kinase domain is preceded by a large unstructured JM region. The collagen-binding trench in the DS domain is shown in red. Collagen binding to this site in wild-type DDR1 induces phosphorylation of cytoplasmic tyrosine residues in both the JM region and kinase domain (shown as yellow circles). None of the mutants are phosphorylated upon collagen incubation. DDR1-W53A has a mutation in the ligand binding pocket in the DS domain. DDR1-R32E and DDR1-L152E are signalling defective mutants with mutations in the ‘signal patch’ region in the base of the DS domain, near the DS-like domain. DDR1-TM1 is a mutant with impaired transmembrane helix association, and DDR1-K655A is a mutant with impaired catalytic function. The locations of mutations are indicated by red stars, and anti-DDR1 epitopes located in the DS-like domain are symbolised by blue and green ovals for wild-type DDR1.

In our previous study, we showed that collagen binding results in redistribution of DDR1 on the cell surface into a more compact structure, and that this collagen-induced clustering can be prevented by a blocking monoclonal antibody (mAb)^10^. We had earlier concluded that the mAb interferes with DDR1 activation allosterically because it binds to an extracellular epitope on the discoidin-like domain (far away from the collagen-binding site on the discoidin domain, see Fig. 1) and does not interfere with DDR1 ligand binding, as assessed by solid phase binding assay of recombinant DDR1 extracellular region to a collagen-mimetic triple-helical peptide^14^. We concluded that collagen-induced clustering is a key step in DDR1 activation, based on the data showing collagen-induced DDR1 redistribution and biochemical evidence of phosphorylation between DDR1 dimers^10^.

### Collagen-induced DDR1 clustering occurs before autophosphorylation

**Figure 2:**
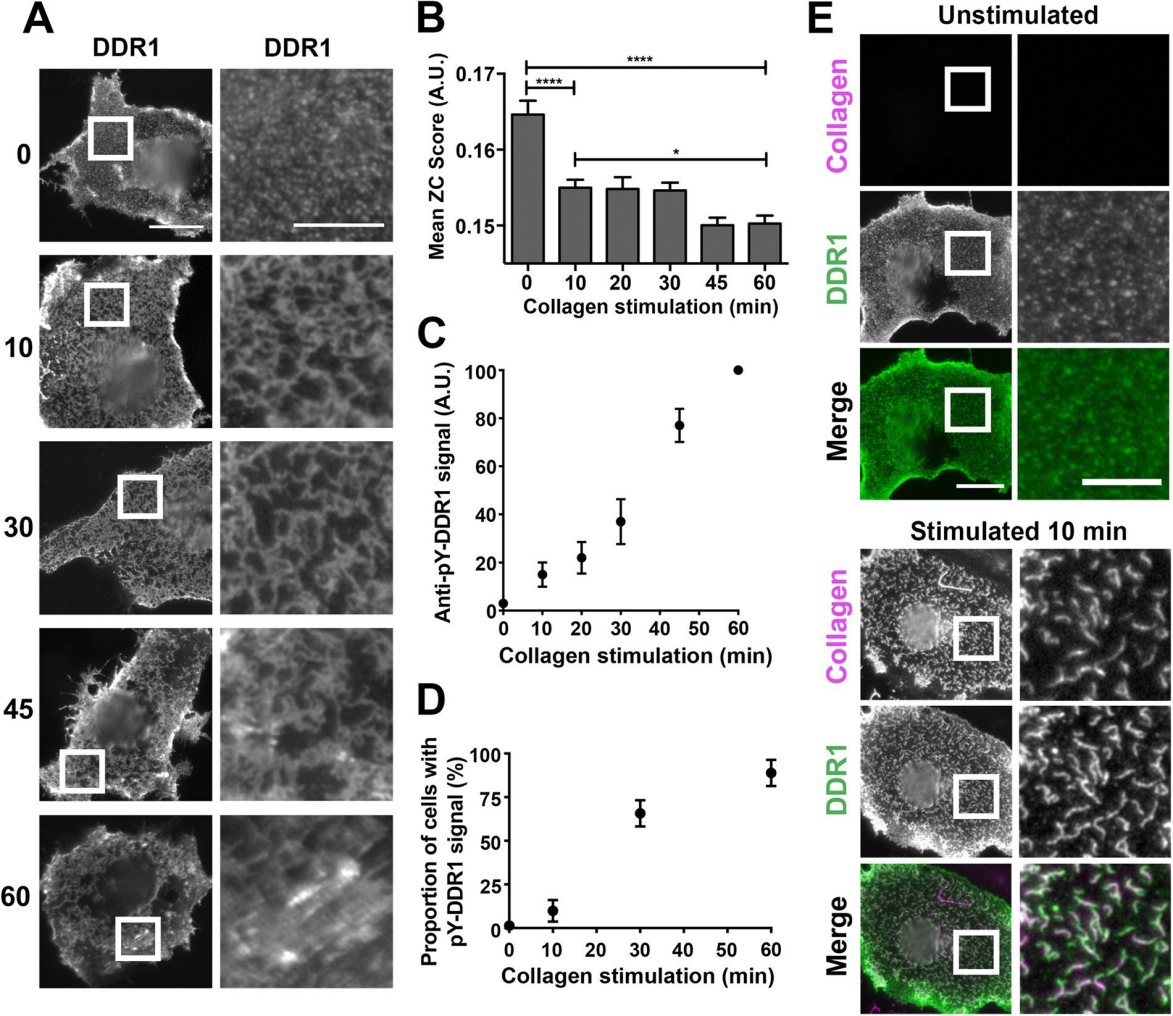
Collagen I induces DDR1 redistribution on the cell surface. COS-7 cells transiently expressing DDR1 were stimulated with collagen as detailed below. (**A**) Cells were stimulated for the indicated times (in minutes) at 37 °C, then incubated on ice with mAb 7A9 against the DDR1 ectodomain, before fixation and secondary Ab staining. (**B**) Cells were stimulated for the indicated times at 37 °C. Staining was done as above. The graph shows mean ZC scores + SEM (N = 200–400 regions from 80–100 cells from 3 independent experiments). *p < 0.05; ****p < 0.0001 (one-way ANOVA, followed by Bonferroni post hoc test). Data are from a different set of experiments than those shown in panel A. (**C**,**D**) Cells were stimulated for the indicated times at 37 °C, then fixed and permeabilised, and immunostained for phospho-tyrosine 513 (pY-DDR1) and for DDR1. (C) Mean pY-DDR1 levels across three experiments: mean values were normalised within experiments and then mean values taken for each stimulation time. N is at least 30 for each stimulation time. (**D**) The proportion of cells expressing DDR1 with pY-DDR1 signal above background levels were manually counted for different stimulation times. Mean percentage values ± SEM (N = 55 for all stimulation times, from two independent experiments). (**E**) Cells were either stimulated with collagen I for 10 minutes at 37 °C or left unstimulated, then incubated on ice with mAb 7A9 against the DDR1 ectodomain (shown in green) and anti-collagen-I mAb (shown in magenta), before fixation, and secondary Ab staining. White boxes in left columns indicate corresponding areas shown at higher magnification in columns to the right. All cells were imaged using a widefield microscope. At least 30 cells were imaged for each condition. Scale bars, 30 μm or 10 μm (enlarged images).

In order to understand how collagen-induced DDR1 redistribution leads to receptor activation, we first performed a time course of collagen stimulation. Stimulation of cells for 5 to 10 minutes with collagen I resulted in DDR1 redistribution from punctate to more compact structures where DDR1 signals were more connected and linear in appearance (Fig. 2A and Supplementary Fig. S1A). These morphologies were maintained over the next 20 minutes or so. Structures with this characteristic morphology were named Collagen-Induced DDR1 Clusters (CIDCs). Incubation times from around 45 minutes showed more irregular structures, with the DDR1 stain seemingly more aggregated in some areas (Fig. 2A, lower two rows).

To assess the degree of clustering in an unbiased way, an automatic quantification of the immunostain signal was undertaken (for detail see Materials and Methods). In brief, an image analysis program was used to quantify the texture of the staining, producing a number called the ZC (Zero crossing of linear density) score, with lower ZC scores indicating a coarser, denser signal. As can be seen in Fig. 2B, collagen-stimulated samples showed a significant decrease in ZC score compared with unstimulated samples. Samples stimulated with collagen for 10–30 minutes showed similar ZC scores, while longer stimulation times resulted in a further drop in ZC scores, indicating further compacting of DDR1 into more dense aggregates.

To show that the CIDC morphology was not dependent on using a particular anti-DDR1 mAb for staining, we used a range of anti-DDR1 mAbs, against four different epitopes^14^, which all detected similar CIDC morphologies (not shown). In addition, an N-terminally Flag-tagged DDR1 construct showed similar structures when immunostained with an anti-Flag Ab (Supplementary Fig. S1B).

As mentioned in the introduction, DDR1 activation, as manifested by autophosphorylation, is a slow process. In transfected HEK-293 cells, it takes about 60–90 minutes for peak phosphorylation induced by collagen I^8^. Since DDR1 phosphorylation kinetics vary in different cell lines and with different incubation conditions (e.g. ref.^15^), we investigated how CIDCs are related to DDR1 phosphorylation in transfected COS-7 cells by staining with phospho-specific Abs against two phosphorylated tyrosines in the kinase activation loop (Abs validated for Western blotting in ref.^10^; Supplementary Fig. S2A,B), or tyrosine-513 in the DDR1b juxtamembrane region (Ab validated for immunostaining and Western blotting in ref.^10^; Supplementary Fig. S2C and Fig. 2C,D). These data clearly show that DDR1 phosphorylation occurs with much slower kinetics than CIDC formation: at 10 minutes stimulation with collagen, when most cells display CIDCs, only 10 ± 4% of cells showed a detectable phosphorylation signal (Fig. 2D), which was very low in comparison with the signals at later time points (Fig. 2C and Supplementary Fig. S3A). Average phosphorylation levels increased over time and, although 66 ± 5% of cells displayed some phosphorylation at 30 minutes of collagen stimulation (Fig. 2D), the average level of phosphorylation roughly doubled from the 30 to the 45 minute stimulation time point (Fig. 2C and Supplementary Fig. S3A).

### Collagen-induced DDR1 clustering is independent of the fibril-forming capacity of the collagen ligand

As expected, CIDCs were positive for collagen I immunostaining (Fig. 2E and Supplementary Fig. S3B). To test whether the CIDC morphology was due to collagen type I forming fibrils, we used a different ligand for DDR1 that is not capable of forming fibrils, collagen type IV^8^. Incubation of cells with collagen IV, induced similar structures to those obtained with collagen I (Supplementary Fig. S3C).

### Signalling-impaired DDR1 mutants do not cluster with collagen stimulation

**Figure 3:**
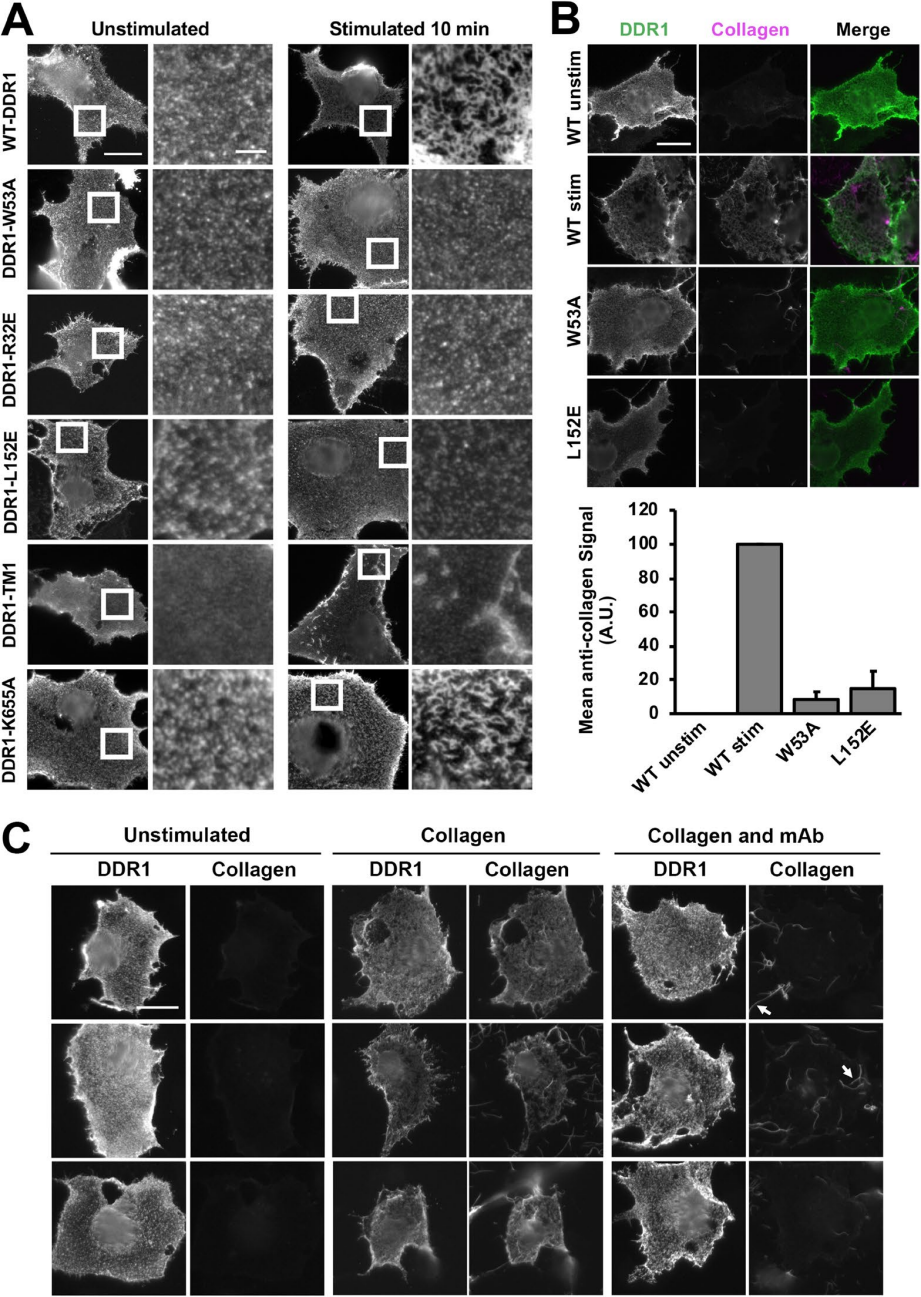
Collagen binding and collagen-induced DDR1 redistribution of signalling defective DDR1 mutants. COS-7 cells transiently expressing WT-DDR1 or the indicated DDR1 mutant were stimulated with collagen I as detailed below. (**A**) Cells were stimulated with collagen for 10 minutes at 37 °C or left unstimulated, then incubated on ice with mAb 7A9, before fixation and secondary Ab staining. White boxes in left columns indicate corresponding areas shown at higher magnification in right columns. (**B**) Cells were stimulated with collagen for 60 minutes at 37 °C or left unstimulated, then incubated on ice with mAb 7A9 (shown in green) and anti-collagen I mAb (shown in magenta), before fixation, and secondary Ab staining. Graph shows the quantified anti-collagen signal with the exclusion of collagen not colocalised with DDR1. The mean collagen-immunostain intensity was calculated for each condition then normalised so that WT-DDR1 collagen-unstimulated and stimulated values were 0 and 100 A.U., respectively. Error bars are SEM (N = 65–121 cells from 3 independent experiments). (**C**) Cells expressing WT-DDR1 were incubated with either collagen I, a mixture of collagen I and anti-DDR1 mAb 7A9, or left unstimulated for 60 minutes at 37 °C. Cells were then immunostained as in B. Arrows indicate collagen aggregates not colocalising with DDR1. Cells were imaged using a widefield microscope. Scale bars, 30 μm or 5 μm (enlarged images in A). For each condition, at least 20 cells were imaged in A, and at least 30 cells were imaged in C.

To show that CIDC formation requires collagen binding, we used a previously characterised DDR1 mutant with a mutation in the collagen binding site of the discoidin domain, DDR1-W53A^10^ (see also Fig. 1). As expected, DDR1-W53A did not form CIDCs upon collagen stimulation (Fig. 3A). Another earlier study identified a different region essential for DDR1 activation termed the ‘signal patch’, which is located in the discoidin domain but far from the ligand binding site^14^. Mutations of key signal patch residues, e.g. R32E or L152E, resulted in signalling-impaired DDR1 that could not be stimulated by collagen to autophosphorylate^14^ (see also Fig. 1). Here, in the absence of collagen, we found DDR1-R32E and DDR1-L152E showed a similar distribution to wild-type DDR1 (Fig. 3A); however, collagen stimulation did not result in CIDCs. This suggests DDR1-R32E and DDR1-L152E do not phosphorylate with collagen stimulation due to their inability to form CIDCs.

### DDR1 clustering is dependent on specific transmembrane helix association and independent of kinase activity

Another signalling impaired mutant, termed DDR1-TM1, has defective transmembrane helix association^5^ (see also Fig. 1). Interestingly, DDR1-TM1 showed a less clustered distribution in the absence of collagen compared with wild-type DDR1 (Fig. 3A and Supplementary Fig. S4A), indicating that transmembrane interactions are important in forming the somewhat aggregated state of unstimulated DDR1 on the cell surface. DDR1-TM1 did not show typical CIDCs, although some collagen-induced changes were observed (Fig. 3A). The final DDR1 mutant we analysed, DDR1-K655A, has an inactivating mutation in the kinase domain^10^ (see also Fig. 1), and showed similar CIDCs as wild-type DDR1 for 10 and 60 minutes of collagen stimulation (Fig. 3A and Supplementary Fig. S4B), indicating that, as expected, CIDC formation is not dependent on kinase activity. This was confirmed by the use of dasatinib, a highly potent DDR1 kinase inhibitor^16–18^. Supplementary Fig. S4C shows that dasatinib treatment did not affect collagen-induced DDR1 redistribution, when cells were stimulated with collagen for 60 minutes.

### Signal patch mutant, DDR1-L152E, is unable to bind to collagen I on the cell surface

We had previously concluded that the signal patch mutants (DDR1-R32E and DDR1-L152E) were not defective in ligand binding, because their isolated ectodomains were able to bind a collagen-mimetic triple-helical peptide in a solid phase assay^14^. However, immunofluorescence experiments showed a lack of collagen binding for DDR1-L152E when expressed in COS-7 cells (Fig. 3B). This was confirmed by flow cytometry (Supplementary Fig. S5A). We also tested DDR1-TM1 which interestingly showed lower levels of collagen binding than wild-type DDR1, despite having an intact collagen-binding discoidin domain (and rest of extracellular region; Supplementary Fig. S5A).

### Function-blocking anti-DDR1 mAb prevents collagen I binding to DDR1 on the cell surface

We next tested collagen binding in the presence of a signal blocking anti-DDR1 mAb and were surprised to observe that collagen binding was inhibited in the presence of the anti-DDR1 mAb (Fig. 3C and Supplementary Fig. S5B). This result was unexpected, since binding of the isolated DDR1 ectodomain to a collagen-mimetic peptide was not inhibited by signal-blocking anti-DDR1 mAbs^14^. Together with the data from Fig. 3B, these results suggest that the inability to bind collagen, of the signal patch mutants and DDR1 wild-type in the presence of anti-DDR1 mAbs, was the underlying reason for the observed absence of CIDCs.

### DDR1 clustering is essential for autophosphorylation induced by a collagen-mimetic peptide

**Figure 4:**
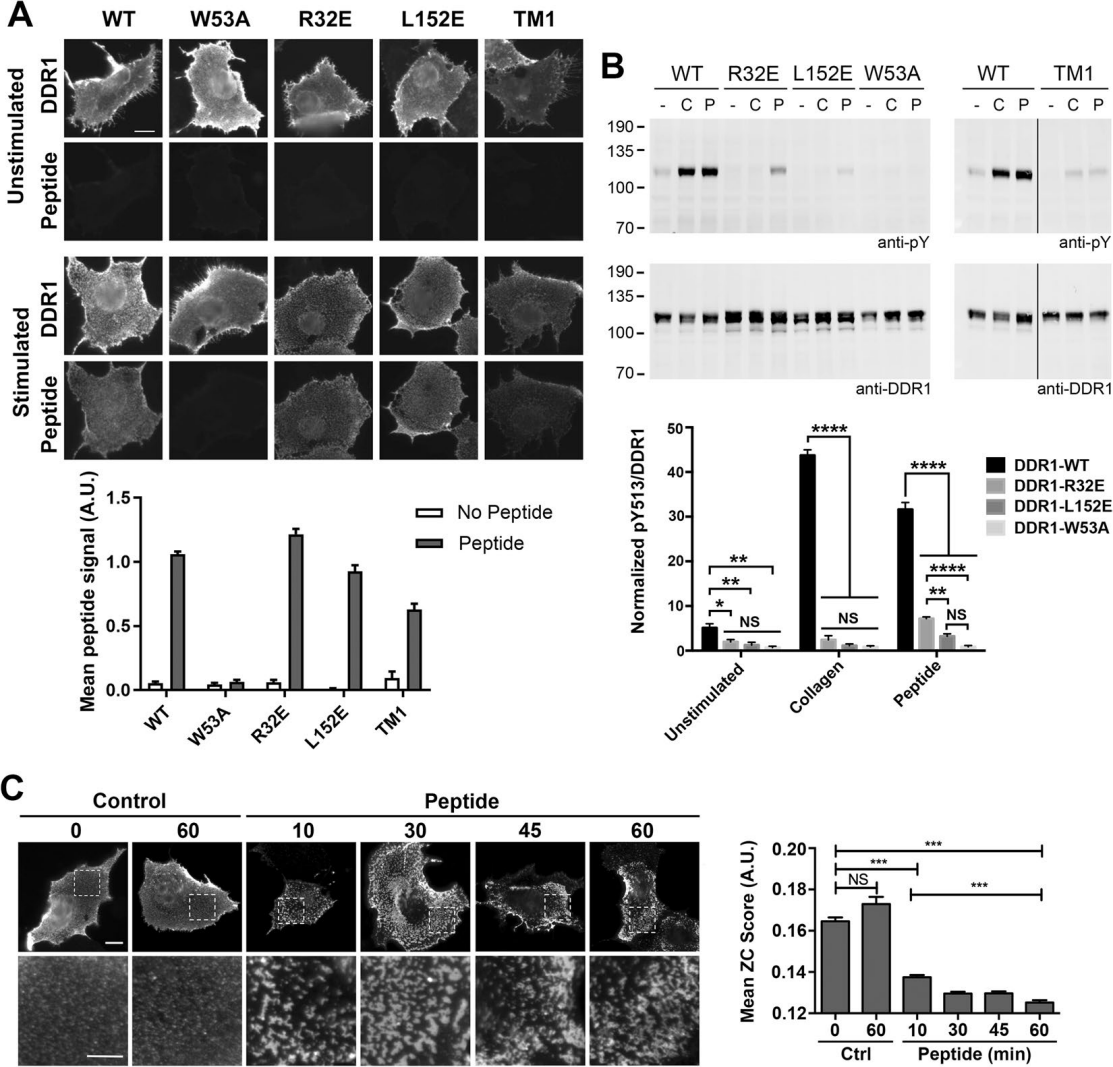
Signalling-defective DDR1 mutants bind triple-helical DDR1 selective peptide but do not phosphorylate with peptide stimulation. (**A**) COS-7 cells transiently expressing wild-type DDR1 or the indicated DDR1 mutant were incubated with or without a biotinylated DDR-selective collagen-mimetic peptide for 60 minutes on ice, followed by incubation with anti-DDR1 mAb 7A9 on ice. Cells were then fixed and incubated with Alexa Fluor-488 goat-anti-mouse IgG and Alexa Fluor-546 conjugated streptavidin. Cells were imaged by widefield microscopy. The graph shows mean fluorescence intensity, normalised to respective DDR1 expression levels. N = 27–31 fields of view from 3 independent experiments. Scale bar, 20 μm. (**B**) HEK293 transiently expressing wild-type DDR1 or the indicated DDR1 mutant were stimulated with collagen I (C), or with DDR-selective collagen-mimetic peptide (P) for 60 minutes at 37 °C or were left unstimulated. Cell lysates were analysed by Western blot using an Ab against phosphorylated Tyr-513 (anti-pY). The blot was stripped and re-probed with anti-DDR1. The positions of molecular mass markers are indicated on the left in kDa. The bar chart shows the densitometry analysis of pY513 band intensities after normalization to total DDR1. Each value is a percentage of the sum of all the pY513/DDR1 signals on the blot. The graph shows mean band intensities + SEM (N = 3). NS, no significance; *p < 0.05; **P < 0.01; ****p < 0.0001 (two-way ANOVA, followed by Tukey’s multiple comparisons test. (**C**) COS-7 cells transiently expressing DDR1 were incubated with collagen-mimetic triple-helical peptide (Peptide) or a control triple-helical peptide without the DDR binding motif (Control) for 0 to 60 minutes at 37 °C, as indicated. Cells were then incubated with anti-DDR1 mAb7A9 Ab on ice, before fixation and incubation with secondary Abs. Lower panels show magnified views of the boxed areas in the upper panels. Cells were imaged by widefield microscopy. Scale bars, 20 μm (upper image) or 10 μm (magnification). Right: ZC score of surface DDR1 staining in cells stimulated with Control or Peptide for 0 to 60 minutes. Data show mean + SEM (N = 200–300 regions from 35–50 cells from 2 independent experiments). NS, no significance; ***p < 0.001 (one-way ANOVA, followed by Bonferroni post hoc test).

While the signal patch mutants did not bind full-length collagen I, they were able to bind a DDR-specific collagen-mimetic triple-helical peptide (Fig. 4A and Supplementary Figs. S5C, S5D), which is in line with the previously published solid phase data^14^. DDR1-TM1 was also able to bind the collagen-mimetic peptide, although at somewhat reduced levels compared with wild-type DDR1. All the constructs that were able to bind the collagen-mimetic peptide exhibited small DDR1 clusters in the presence of peptide (Fig. 4A). Despite being able to bind the collagen-mimetic peptide, the signal patch mutants and DDR1-TM1 were defective in peptide-induced autophosphorylation, with only DDR1-R32E showing a very low phosphorylation signal (Fig. 4B).

We next used immunofluorescence to investigate whether peptide-induced autophosphorylation was correlated with DDR1 redistribution on the cell surface. As shown in Fig. 4C, collagen-mimetic peptide induced DDR1 clustering at 37 °C; however the morphology was different to typical CIDCs, with more rounded and less connected structures observed. Similar to collagen-induced DDR1 redistribution, peptide-induced DDR1 redistribution did not require kinase activity as it occurred to similar extents when cells were incubated with the kinase inhibitor dasatinib (Supplementary Fig. S6A).

Despite the fact that the signal-patch mutants and DDR1-TM1 were able to bind the collagen-mimetic peptide, this peptide was not able to induce DDR1 redistribution for these DDR1 mutants (Supplementary Fig. S6A). This result suggests that receptor-receptor contacts may be required for peptide-induced DDR1 clustering and that peptide-induced clustering is a necessary step in DDR1 phosphorylation.

### Collagen-induced DDR1 phosphorylation occurs in two stages

**Figure 5:**
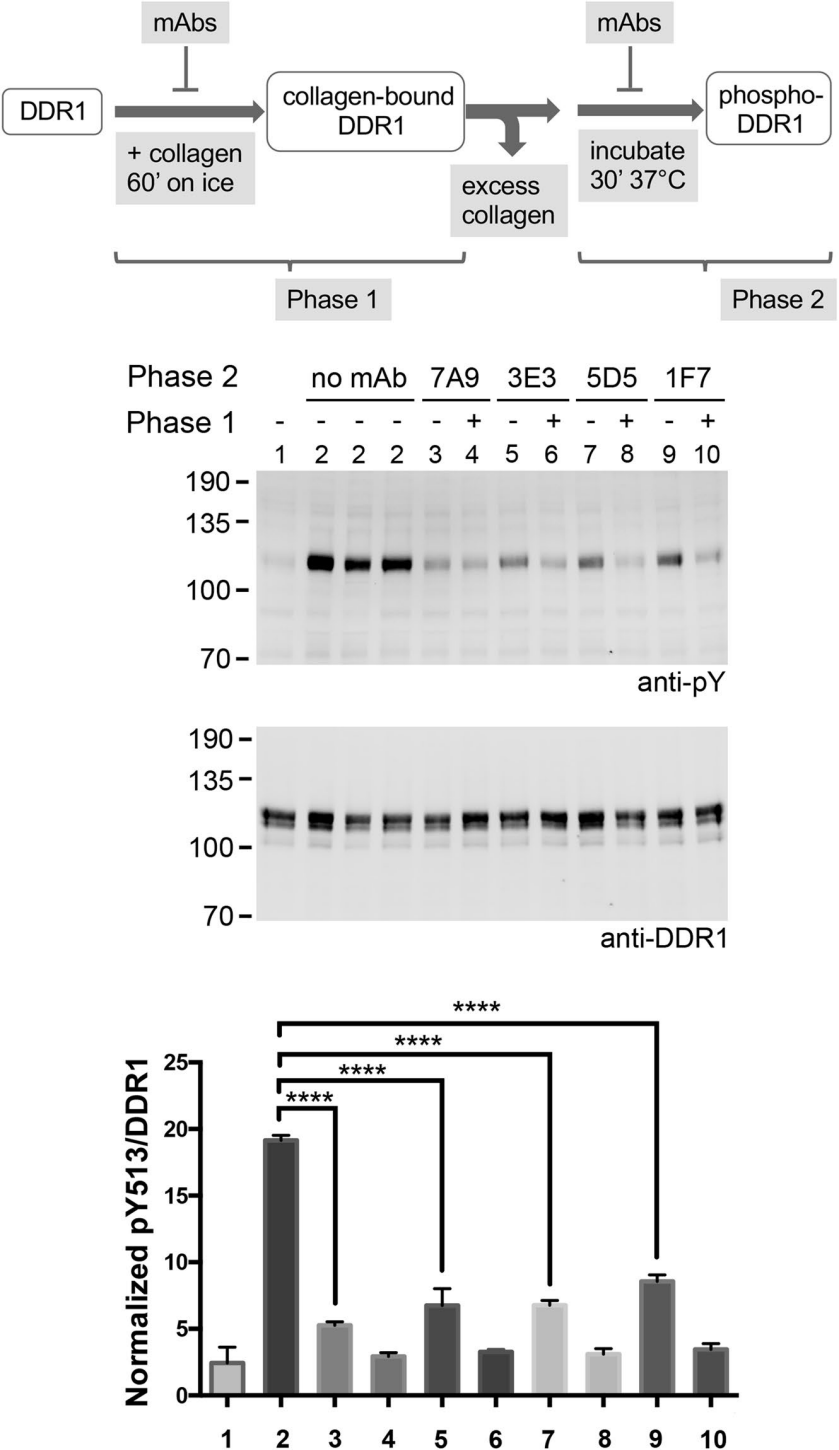
Anti-DDR1 mAbs block phosphorylation of collagen-bound DDR1. The diagram at the top gives an overview of the experimental procedures. HEK293 cells transiently expressing wide-type DDR1 were first incubated with collagen I for 60 minutes on ice, in the presence (+) or absence (−) of the indicated anti-DDR1 mAbs (Phase 1). Following washes, cells were incubated for 30 minutes at 37 °C, in the absence (no mAb) or presence of the indicated mAbs (Phase 2). The sample shown in lane 1 was lysed immediately after the incubation with collagen on ice. Samples labelled 2 were replicate lysates from 3 different wells. Cell lysates were analysed by Western blot using a mAb against phosphorylated Tyr-513 (anti-pY). The blot was stripped and re-probed with rabbit anti-DDR1. The positions of molecular mass markers are indicated on the left in kDa. The bar chart shows the densitometry analysis of pY513 band intensities after normalization to total DDR1. Each value is a percentage of the sum of all the pY513/DDR1 signals on the blot. The graph shows mean band intensities + SEM (N = 3). ****p < 0.0001 (one-way ANOVA, followed by Dunnett's multiple comparison test).

As shown in Supplementary Fig. S1, Fig. 2 and Supplementary Fig. S3, CIDC structures appear quickly, much earlier than the majority of DDR1 phosphorylation signals. We thus hypothesised that DDR1 phosphorylation is a process that happens in at least two stages, with the first one involving CIDC formation and the second one consisting of further DDR1 aggregation to allow kinase activation in dense molecular clusters. In order to address whether anti-DDR1 mAbs could block DDR1 phosphorylation after CIDC formation, we designed experiments that allowed uncoupling of collagen binding from the second step. Incubation of cells with collagen I on ice for 60 minutes led to similar CIDC structures as stimulation with collagen I at 37 °C for 10 minutes (Supplementary Fig. S6B). Further incubation of cells for a period at 37 °C in the absence of collagen (and following washes), allowed phosphorylation to occur to similar extents as is routinely recorded with continuous stimulation with collagen (Fig. 5). When function-blocking mAbs were present during the incubation at 37 °C, collagen-induced phosphorylation was inhibited (Fig. 5), demonstrating that anti-DDR1 mAbs can interfere with the second step of DDR1 activation.

### Structured illumination microscopy reveals correlation of DDR1 phosphorylation with high density DDR1 aggregation

**Figure 6:**
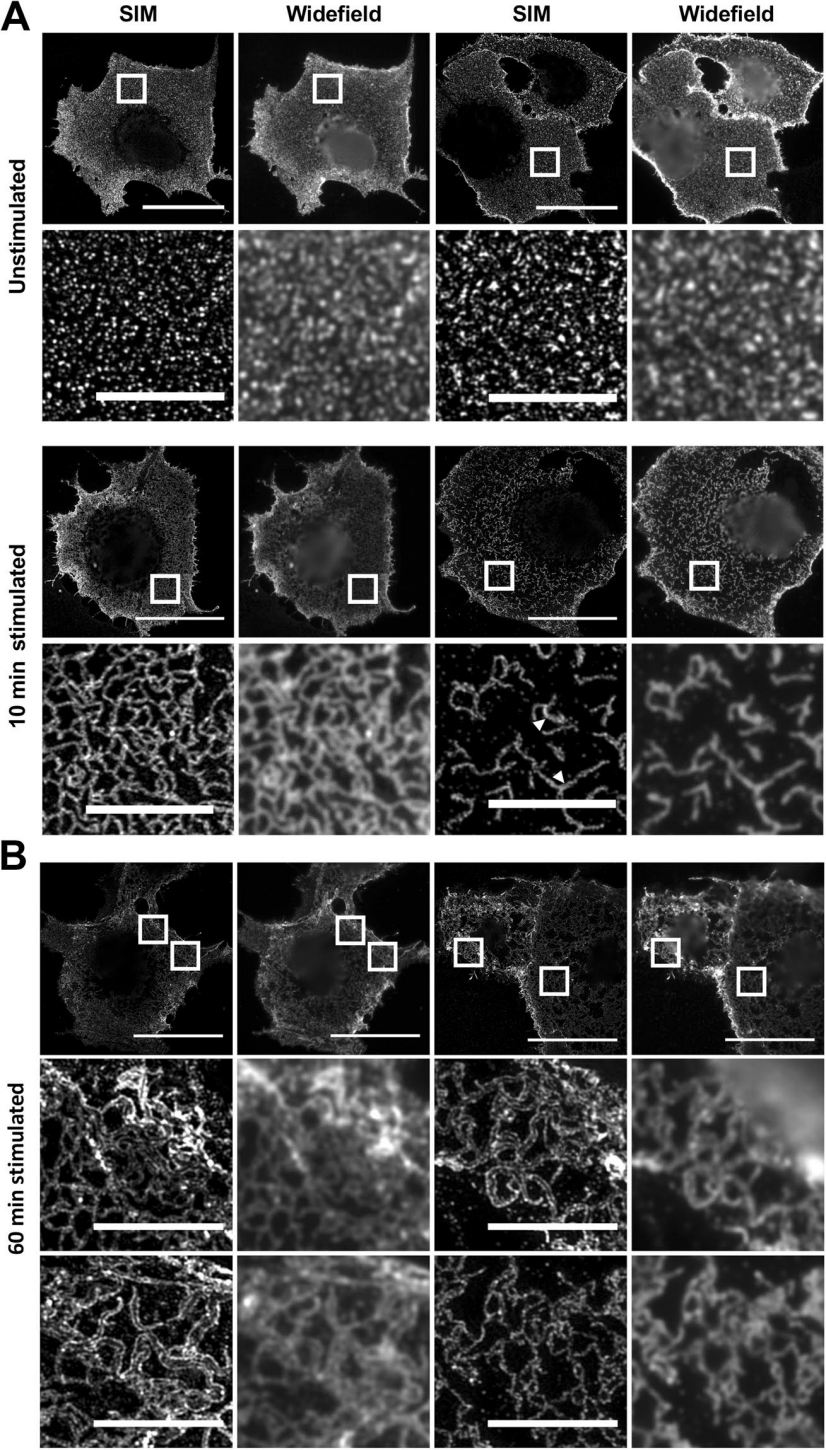
SIM images reveal DDR1-immunostain double-walled structures for cells stimulated with collagen for 60 minutes at 37 °C. COS-7 cells transiently expressing DDR1 were stimulated with collagen I for 10 or 60 minutes at 37 °C or left unstimulated, then incubated on ice with mAb 7A9, before fixation and secondary Ab staining. 3D-SIM images were acquired using a Zeiss ELRYA microscope; images are from one of 15 slices. Lower panels show magnified views of the boxed areas in the upper panels. Arrowheads in A indicate example structures wider than 200 nm (full width at half maximum). Scale bars, 30 μm (upper panels) or 5 μm (magnified images). At least 10 cells were imaged for each condition.

As shown in Fig. 2A, at 60 minutes of continuous collagen incubation DDR1 appears more aggregated than at earlier time points and this is correlated with increased DDR1 phosphorylation. In order to explore the aggregated state in more detail, we used structured illumination microscopy (SIM), a super-resolution technique particularly suited to samples with dense networks made up of fine structures^19^. SIM provides a lateral resolution up to twice that achievable with diffraction-limited wide-field microscopy (typically ~110 nm when imaging cells). For unstimulated samples, the roughly circular puncta with DDR1 immunostain appeared similar in wide-field microscopy and SIM (Fig. 6A). Cells stimulated with collagen for 10 minutes also displayed similar structures in SIM and widefield microscopy, with a little more detail revealed in SIM. Measuring the width of the linear structures and the diameter of the puncta showed them to be mostly ~110 nm across (defined by the full width at half maximum intensity), which is at the limit of the microscope's resolution; hence the puncta and linear structures are 110 nm or smaller across, with some linear structures at 10 minute collagen stimulation showing widths of around 200 nm (see arrowheads in Fig. 6A).

For cells stimulated with collagen for 60 minutes, SIM revealed additional detail not apparent from the widefield images: DDR1 was found in linear structures with no signal in the middle; these structures appear as ‘double-walled’ structures (Fig. 6B). These double-walled structures were observed across many cells and most often located towards the cell periphery where the DDR1 stain appeared most aggregated in the widefield images (e.g. in both of the upper high magnification images in Fig. 6B); the magnified field in the lower right shows an area from a more medial location in the cell and does not show double-walled structures but structures similar to what is observed at 10 minute collagen stimulation. Double-walled structures were also observed in images stained with an anti-DDR1 mAb against a different epitope (Supplementary Fig. S7A).

**Figure 7:**
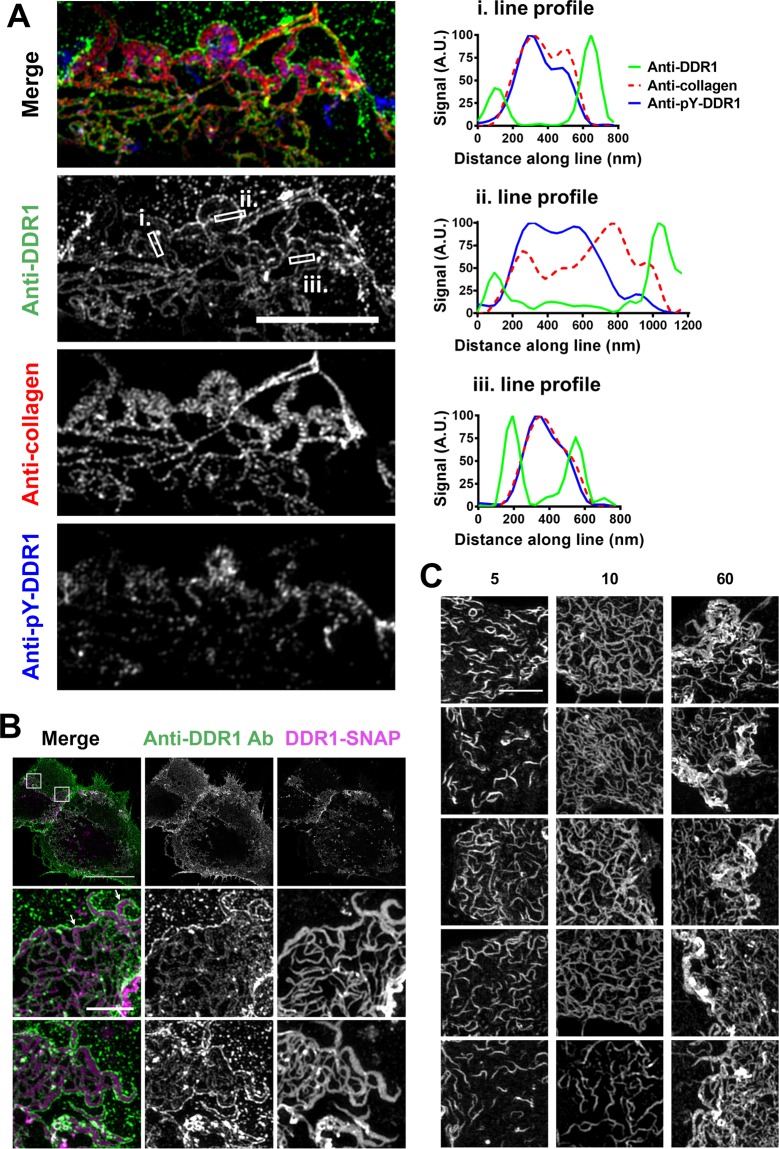
Aggregated and phosphorylated DDR1 is present in the double walled anti-DDR1 structures. (**A**) COS-7 cells transiently expressing DDR1 were stimulated with collagen I for 60 minutes at 37 °C, then incubated on ice with anti-DDR1 mAb 7A9 and anti-collagen I mAb, before fixation, permeabilisation and immunostaining for phospho-tyrosine 513 (pY-DDR1). Intensity of the three stains was measured across the three lines shown (with a line width of 200 nm), the data were normalised so that the lowest and highest value from each stain was 0 and 100 A.U. (**B**,**C**) COS-7 cells transiently expressing DDR1-SNAP were incubated with SNAP-Surface Alexa Fluor-546 for 60 minutes at 37 °C, then stimulated with collagen I for 60 minutes (**B**) or for 5, 10, or 60 minutes (**C**) at 37 °C. Cells were then incubated on ice with anti-DDR1 mAb 5D5, before fixation, and secondary Ab staining (**B**), or fixed and mounted (**C**). 3D-SIM images were acquired using a Zeiss ELRYA microscope. Images are from a maximum intensity projection of all 15 slices (**B**) or from a single slice (**A**,**C**). White boxes indicate corresponding areas shown at higher magnification in lower images (**B**). Scale bars, 5 μm (**A**), 30 μm (upper image in B), 2 μm (enlarged images in B) or 3 μm (**C**). White arrows indicate examples of anti-DDR1 mAb binding at the edges of aggregated DDR1-SNAP signal (**B**). At least 10 cells were imaged for each condition.

To investigate the double-walled structures further, cells were co-stained with Abs against DDR1, collagen I, and a phospho-specific anti-DDR1 Ab against phospho-trosine-513 (anti-pY-DDR1, previously characterised in ref.^10^). While anti-DDR1 showed the familiar double-walled structures, neither collagen nor pY-DDR1 stains were distributed in the same way (Fig. 7A). In fact, collagen and pY-DDR1 filled the ‘gap’ of the double-walled structures, which is demonstrated by the line profiles in Fig. 7A. Supplementary Fig. S7B shows similar profiles from five different cells, again demonstrating anti-pY and anti-collagen I signals peaking in the trough of the anti-DDR1 signal. It is also clear that the highest pY signals are seen in cellular regions that contain the double-walled structures. Cellular regions not containing double-walled structures have much lower pY signals (e.g. lower left area in bottom row images, Supplementary Fig. S7B).

Since anti-pY-DDR1 signals appeared highest in the regions of low anti-DDR1 signals, we hypothesised that the ‘gap’ regions contain DDR1 molecules that are highly aggregated and that the anti-DDR1 epitopes on the discoidin domain may no longer be accessible to the mAbs in the most aggregated regions. We therefore investigated DDR1 activation without anti-DDR1 mAbs. To this end, we created SNAP-tagged DDR1 and used an Alexa Fluor dye to covalently label the SNAP tag. The SNAP-tagged DDR1 had the tag inserted into the highly flexible extracellular juxtamembrane region, where we previously inserted flexible segments without affecting collagen-induced DDR1 phosphorylation^7^. DDR1-SNAP was expressed on the cell surface and could be stimulated to autophosphorylate with collagen I similar to untagged DDR1 (Supplementary Fig. S8A,B).

To investigate whether the double-walled structures contained DDR1 molecules in the ‘gaps’, we stimulated cells expressing DDR1-SNAP with collagen for 60 minutes, then double stained with a SNAP surface label and anti-DDR1. The resulting images showed that indeed, the double-walled structures contain the SNAP label in the ‘gaps’, indicating highly aggregated DDR1 to be present in these regions that lack anti-DDR1 stain (Fig. 7B). Figure 7B also shows anti-DDR1 signal apparent at the edges of the SNAP signal.

DDR1 aggregation over time was also observed in SNAP-tagged DDR1 imaged at 5, 10 and 60 minutes of collagen stimulation, with the 60 minute timepoint clearly showing highly aggregated signals (Fig. 7C). Additionally, the images from the 10 minute time point showed somewhat wider structures than at the 5 minute timepoint. Furthermore, phosphorylated DDR1 was often correlated with the brightest and most aggregated DDR1-SNAP structures in cells stimulated with collagen for 60 minutes (Supplementary Fig. S8C). Using N-terminally tagged Flag-DDR1 and anti-Flag staining additionally showed aggregated structures at 60 minute collagen stimulation that did not have the ‘double-walled’ morphology, indicating that the Flag epitope is accessible in aggregated DDR1 (Supplementary Fig. S8D).

**Figure 8:**
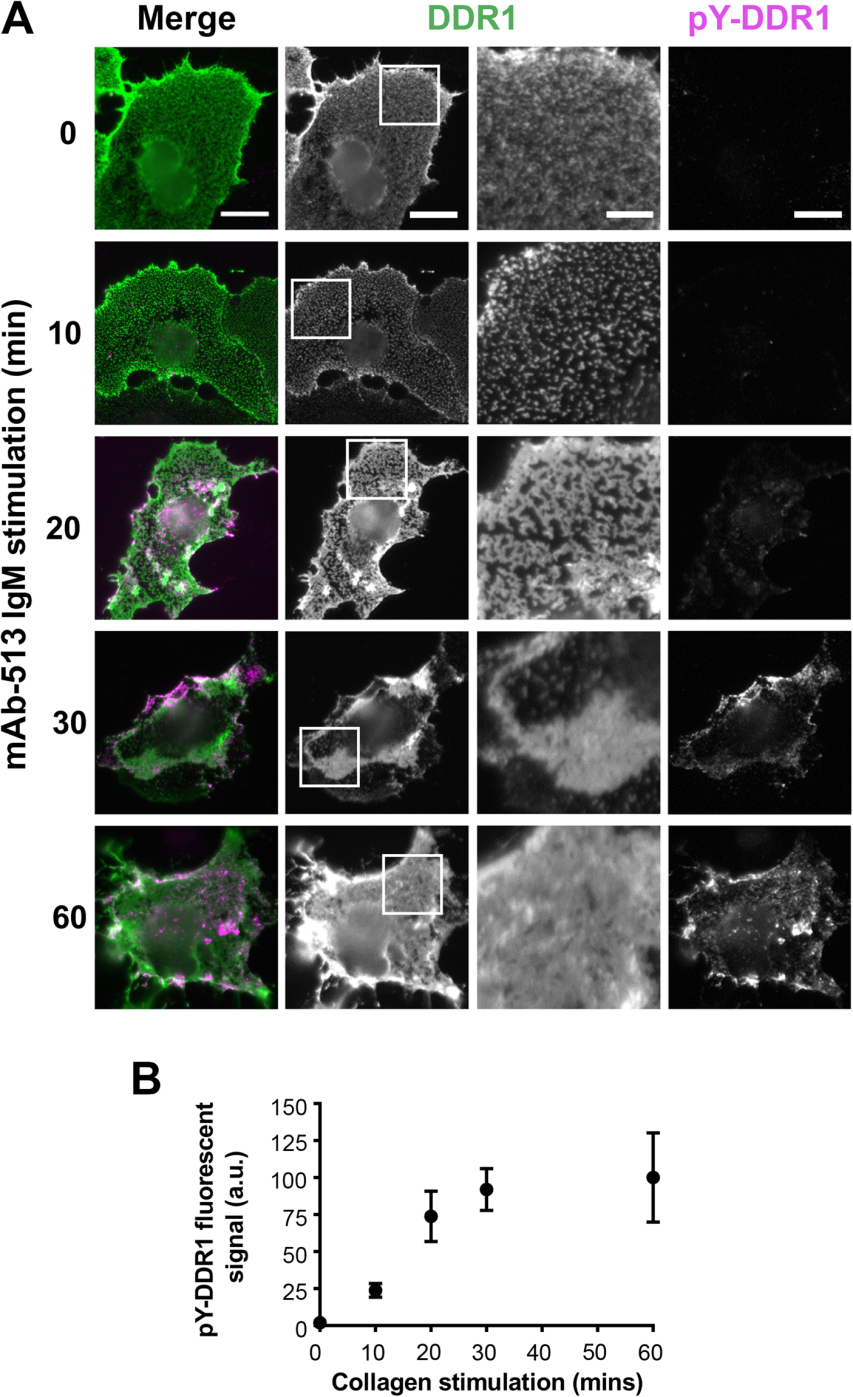
mAb-513-induced DDR1 redistribution and phosphorylation. (**A**) COS-7 cells transiently expressing DDR1 were either incubated with mAb-513 IgM for the indicated times at 37 °C or left unstimulated, then incubated on ice with anti-DDR1 mAb 7A9, before fixation, permeabilisation and immunostaining for phospho-tyrosine 513 (pY-DDR1). Cells were imaged using a widefield microscope. White boxes in DDR1 images indicate corresponding areas shown at higher magnification in images to the right. Scale bars, 30 μm or 10 μm (magnification). (**B**) Cells were treated and imaged as above. Mean pY-DDR1 signal for each cell was calculated and averaged for the stimulation time. Values were normalised so that the mean value for 0 and 60 minutes was 0 and 100 A.U respectively. Error bars are SEM. N = 15–23.

DDR1 phosphorylation can also be induced by an activating, multimeric IgM Ab, mAb-513 (see ref.^10^). Ligand-independent stimulation with this Ab resulted in time-dependent aggregates that were correlated with phosphorylation (Fig. 8). Altogether, our results show that DDR1 autophosphorylation is a result of DDR1 aggregation into dense clusters.

## Discussion

Here we show that DDR1 activation occurs in two phases: a rapid (5–10 minute) redistribution into morphologically distinct structures, followed by slower (45–60 minute) compaction into dense aggregates. The initial clusters contain unphosphorylated DDR1, demonstrating receptor-ligand ligation is not sufficient for DDR1 activation. The local concentration of DDR1 dimers in these loosely packed clusters may not be high enough to initiate kinase activity; kinase activity is only triggered once DDR1 has aggregated into dense clusters. In our previous report we showed that collagen binding induces DDR1 autophosphorylation between neighbouring DDR1 dimers^10^, a process that can only occur if DDR1 phosphorylation takes place in densely packed structures. Here we provide evidence that DDR1 autophosphorylation is correlated with a time-dependent DDR1 localisation to structures with high density. This suggests that kinase activity is only stimulated once a certain molecular density is reached. Such a density-driven signalling mechanism is not without precedent: triggering of T-cell receptor-CD3 complexes only occurs in dense clusters^20^. Here we show DDR1 localisation to densely packed clusters is independent of kinase activity, ruling out the formation of signalling clusters as a consequence of DDR1 phosphorylation.

This study gives a simple mechanistic explanation for the unusually slow process of ligand-induced DDR1 phosphorylation. Previously, it was speculated that other accessory proteins may be required to allow DDR phosphorylation to occur (discussed in ref.^3^). While we cannot rule out that such proteins regulate DDR activation, our model does not require them. Interestingly, the tetraspanin TM4SF1 was found to be required for collagen-induced DDR1 clustering in cancer cells, but this clustering process did not result in DDR1 autophosphorylation, and instead led to non-canonical DDR1 signalling, independent of its kinase activity^11^. This study highlights the importance of understanding how DDR1 clustering in different contexts can affect diverse signalling pathways.

DDR1 redistributed on the cell surface when stimulated with a range of different ligands. The morphology of these structures was somewhat different, depending on the ligand (collagen I, collagen IV, collagen-mimetic peptide, or the multimeric anti-DDR1 mAb 513), suggesting different signalling outcomes. However, little is known about how DDR1 signalling is modulated by different collagen types. Various collagenous ligands are known to induce different degrees of receptor clustering for the platelet collagen receptor GPVI^21^. For other RTKs, ligands that lead to different cellular responses were recently shown to affect the strength of dimers (EGFR) or differential clustering (FGFR1)^22,23^.

Our study gives new insight into ligand binding to DDR1 on the cell surface. We found DDR1 binding to full-length collagen requires a region on the discoidin domain outside the ligand binding pocket, previously called the “signal patch” that has been suggested to be a secondary collagen binding site^14^. The signal patch was not necessary for DDR1 binding to a collagen-mimetic DDR-selective peptide, however the signal patch was required for peptide-induced DDR1 clustering and phosphorylation. These data indicate that ligand-induced DDR1 clustering requires receptor-receptor interactions potentially at the signal patch.

While other studies have observed collagen-induced DDR1 clustering^6,11–13^, these reports did not address the mechanism that triggers DDR1 kinase activation. Early work by Mihai *et al*. established collagen-induced DDR1 clustering within 5 minutes of ligand addition to DDR1b-YFP^6^. A more recent study by the same group observed collagen-induced clustering of DDR1b-YFP in a pre-osteoblast cell line at 30 minutes and 4 hours after collagen addition. While ligand-induced clustering was clearly seen at the 30 minute stimulation time, curiously, there was little to no colocalisation of DDR1-YFP with collagen I at this timepoint and only a sub-population of clusters was positive for collagen I at the 4 hour time point^13^. The authors attribute lack of colocalization with collagen to the fact that DDR1b may have rapidly internalized (with the bound ligand) following collagen binding. Collagen-induced DDR1 endocytosis has been described by two groups^6,24^. While our studies confirmed rapid collagen-induced DDR1 clustering on the cell surface (this study and ref.^10^), we have not been able to reproduce the data on collagen-induced DDR1 internalisation (not shown). When DDR1 was stimulated with collagen immobilized on beads, DDR1 was recruited to the beads and phosphorylation was observed only at the DDR1/bead contact area on the cell surface^10^, indicating that endocytosis is not required for DDR1 phosphorylation. Clustering of DDR1b-YFP was also observed under different conditions, where mouse GD25 cells were cultured on fibrillar collagen and, similar to the results in the present study, initial cluster formation was independent of DDR1 kinase function^12^.

Ligand-induced clustering (higher-order multimerisation) has been observed for other RTK families: it is a requirement for Eph receptor kinase activation^25^, and is thought to modulate EGFR signalling^26–28^. DDR1 kinase activation shares with Eph receptors the requirement for clustering, although DDR1 kinase activity requires specific transmembrane contacts^10^. In agreement with this, we found here specific transmembrane helix associations are needed for DDR1 to reorganise on the cell surface. These transmembrane associations are also required for DDR1 to adopt the somewhat clustered arrangement in the absence of ligand. This further reinforces the concept that individual DDR1 dimers are associated via their transmembrane region into groups of dimers, even in the absence of ligand stimulation.

Here we have dissected the process of DDR1 activation into two stages: an initial phase of ligand binding and spatial reorganisation, followed by aggregation of DDR1 into dense signalling clusters. If anti-DDR1 mAbs are added during the second phase, after collagen binding, they block phosphorylation of ligand-bound DDR1, hence are likely preventing DDR1 from compacting into dense clusters. This suggests that extracellular receptor-receptor contacts may be required for DDR1 aggregation into dense clusters. Alternatively, it is possible that the anti-DDR1 mAbs block further aggregation simply by sterically preventing DDR1 molecules moving closer together, in which case extracellular receptor-receptor contacts would not strictly be required for formation of dense signalling clusters. The second possibility is supported by our previous observation that DDR1 dimers lacking an extracellular region are recruited to DDR1 signalling clusters^10^.

In conclusion, we show that DDR1 phosphorylation is correlated with localisation to dense signalling clusters. Future studies are needed to obtain more quantitative mechanistic understanding, for example super-resolution imaging methods to analyse the number of DDR1 molecules in different types of clusters. This may determine a threshold of molecular density that initiates DDR1 kinase activity, similar to T-cell receptor triggering^20^. It is also not clear whether DDR1 autophosphorylation occurs due to a certain molecular density and/or a particular number of DDR1 molecules within the cluster. In this context, a recent study found Eph receptor monomers must cluster into 6–8-mers before further clustering into hundreds of Eph receptors can occur^29^. More detailed super-resolution imaging is also required to define the ratio of collagen molecules to DDR1 molecules in signalling clusters. Such data would reveal the relationship between ligand occupancy and DDR1 kinase activation. Our present study has defined a density-driven signalling mechanism that explains the unusually slow DDR1 activation kinetics and provides a foundation upon which the details of DDR1 molecular assembly can be built, while also providing a potential drug design strategy of inhibiting DDR1 by blocking its assembly into dense clusters.

## Materials and Methods

### Cell culture

Cell culture was carried out as in our previous work (e.g refs.^5,7,10^). Monkey COS-7 fibroblast-like kidney cells and human embryonic kidney (HEK) 293 cells were obtained from ATCC, Manassas, VA. Cells were grown at 37 °C, 5% CO_2_, in Dulbecco's modified Eagle's medium/F12 (DMEM/F12) nutrient mixture with 10% foetal bovine serum, 2 mM L-glutamine, 100 units/ml penicillin, 100 µg/ml streptomycin. Cells were tested for mycoplasma contamination every 4 months and were confirmed negative.

### Chemicals and reagents

Collagen I (acid-soluble from rat tail; C-7661) and collagen IV (acid-soluble from placenta; C-5533) were from Sigma (Gillingham, UK). The triple-helical collagen-mimetic peptides, (GPP)_5_-GPRGQOGVNleGFO-(GPP)_5_GPC-NH_2_ (DDR-selective peptide); biotin-(GPP)_5_-GPRGQOGVMGFO-(GPP)_5_GPC-NH_2_ (biotinylated DDR-selective peptide), GPC(GPP)_10_-GPC-NH_2_ (control peptide) were obtained from Prof. Richard Farndale, University of Cambridge, UK. The peptides were synthesized by Fmoc (*N*-(9-fluorenyl)methoxycarbonyl) chemistry as a C-terminal amide on TentaGel R RAM resin in an Applied Biosystems Pioneer automated synthesiser and purified as described^30^.

Bovine serum albumin (BSA) was from Fisher Scientific (Loughborough, UK). SNAP surface Alexa Fluor-546 and SNAP Surface Block were from New England Biolabs, UK. Streptavidin Alexa Flour-546 was from Invitrogen. Dasatinib was from Selleckchem, UK.

The following primary Abs were used: rabbit anti-DDR1 (SC-532, Santa Cruz, Dallas, TX; for Western blotting); monoclonal rabbit anti-phospho-DDR1 (Tyr513, E1N8F, Cell Signaling; for Western blotting and immunostaining, validated in ref.^10^); polyclonal rabbit anti-phospho-DDR1 (Tyr 513, abx012650, Abbexa; for immunostaining); rabbit anti-phospho-DDR1 (Tyr 792, 11994, Cell Signaling, validated in ref.^10^); rabbit anti-phospho-DDR1/DDR2 (DDR1 Y796, DDR2 Y740, MAB25382, R&D Systems, validated in ref.^10^); mouse anti-Flag IgG1 clone M2 (Sigma); mouse anti-rat collagen type I (clone 1F10C2, IgG2b; Chondrex). Mouse anti-DDR1 mAbs, 7A9, 3E3, 5D5 and IF7, were generated in our lab^14^. The multimeric anti-DDR1 IgM Ab (mAb-513), a gift from Dr Michel Faure, SUGEN Inc., was used in the form of ascites. Secondary Abs were as follows: goat anti-rabbit Ig-horseradish peroxidase conjugated (P0448, DAKO A/S, Denmark); sheep anti-mouse Ig-horseradish peroxidase (Amersham Biosciences, Little Chalfont, UK); goat anti-mouse IgG Alexa Flour-488 (Invitrogen); goat anti-mouse IgG1 Alexa Fluor-488 (Invitrogen); goat anti-rabbit Alexa Fluor-546 (Invitrogen); goat anti-mouse IgG2b Alexa Fluor-555 (Invitrogen); goat anti-rabbit IgG Alexa Fluor-647 (Invitrogen); goat anti-mouse IgG FITC-conjugated (F-9006, Sigma).

### DNA constructs and site-directed mutagenesis

All DDR1 expression plasmids contained the cDNA of the DDR1b isoform of DDR1, cloned into the mammalian expression vector pRK5 (BD Pharmingen). The following mutants were generated previously: DDR1-TM1 (DDR1b-L430G/L431P)^5^; DDR1b-R32E and DDR1b-L152E^14^; DDR1b-W53A and DDR1b-KD (DDR1b-K655A)^10^. Flag-DDR1, containing an N-terminal Flag tag followed by a short linker, and DDR1-SNAP were generated by the FastCloning method^31^. The template for DDR1b was wild-type DDR1b cloned into the pSP72 vector (Promega). For SNAP-DDR1, cDNA encoding the SNAP-tag (pSNAPf plasmid, NEB, UK) was fused with DDR1b cDNA. The cDNAs encoding Flag-DDR1 and DDR1-SNAP were subcloned into pRK5, after verifying correct sequences by DNA sequencing. The DDR1-SNAP construct encoded DDR1b in which the SNAP tag is inserted after glycine-378. This site was chosen for the insertion as previous work showed that insertion of a flexible region at this site did not affect collagen-induced DDR1 activation^7^. Primers used for generating the mutant constructs are available on request.

### Transfection of cells

COS-7 cells were grown on coverslips in 24-well tissue culture plates (for fluorescence imaging) or in 6-well plates (for flow cytometry) and transfected with relevant DDR1 expression plasmids using Fugene HD (Promega, Madison, WI) or Lipofectamine 3000 (ThermoFisher Scientific, UK) according to the manufacturers’ instructions. HEK293 cells were grown in 24-well tissue culture plates and transfected with relevant DDR1 expression plasmids by calcium phosphate precipitation, as described^32^.

### Immunofluorescence staining

Transfected COS-7 cells were incubated with collagens (at 10 μg/ml or 20 μg/ml), DDR selective peptides (at 50 μg/ml) or other chemicals as detailed in the Figure legends, in phenol-red free DMEM/F-12 (Gibco, ThermoFisher Scientific, UK). Collagens were diluted from 1 mg/ml stock solutions in 100 mM acetic acid. Wells that were not stimulated with collagens contained the same concentration of acetic acid as collagen-containing wells. To label DDR1 exclusively on the cell surface, cells were incubated on ice with the primary Abs diluted in 4% (w/v) BSA/phosphate buffered saline (PBS), pH = 7.4, followed by washing with ice-cold PBS and fixation with 4% paraformaldehyde/PBS for 15 minutes at room temperature. To immunostain intracellular epitopes, cells were then permeabilised in 0.1% Triton-X100 in BSA/PBS for 10 minutes. Cells were blocked in BSA/PBS before incubation with secondary Abs in BSA/PBS. If surface staining was not required, cells were fixed immediately after stimulation, then blocked and permeabilised if needed. After staining, cells were washed in PBS and distilled water, followed by mounting onto microscopy slides with ProLong Gold Antifade reagent (Invitrogen).

### SNAP-tag labelling

Transfected COS-7 cells were incubated with 5 μM SNAP surface label, diluted in phenol-red free DMEM/F12 at 37 °C for one hour, followed by washing and incubation with 20 μM SNAP-Surface Block in complete medium for 20 minutes at 37 °C. Cells were then stimulated and immunostained as described above.

### Microscopy

Widefield microscopy was carried out on an Olympus BX51 microscope. SIM was carried out on a Zeiss ELYRA microscope. The SIM image was calculated from five grating-angles and five phase shifts. A z-stack was acquired using 15 slices covering 1.65 μm axially. The raw images were processed into super-resolution images using the Zeiss ZEN (Black Edition) software with the default settings. To correct for chromatic shifts in the colour channels a z-stack was acquired of sub-diffraction multicolour fluorescent beads, using the same microscope set-up. The images were checked for any potential artefacts from SIM reconstruction as detailed in^33^.

### Image analysis using ImageJ

Automated measuring of collagen or phosphorylated DDR1 immunostains of individual cells was done by firstly manually cropping each image and then segmenting cells with a mask covering the entirety of the cell and not including any areas outside the cell. This process was carried out using the Fiji distribution of ImageJ^34^. A mask of the cell was created from the DDR1 immunostain signal, and pixels were selected that had a greater intensity than a pre-determined background level. To ensure all of the cell was selected the “dilate” ImageJ process was run to add pixels to the edges of objects which helps to create enclosed areas which were filled in with the “fill holes” process, followed by the “erode” process that removes pixels from the edges to return the mask close to the original outline. This process was repeated twice to ensure the entire cell was selected by the mask. The mask was then applied to the different channels to measure the mean intensity of the immunostains for the entirety of the cell. To quantify phosphorylated DDR1 immunostain the mean signal value was calculated for the entirety of each cell (using the aforementioned masks), and then the mean value was taken for all the cells within the time point of collagen stimulation. To quantify collagen immunostain intensity, cells were further manually cropped to remove areas of the image where collagen did not colocalise with the DDR1 immunostain. The rest of the process was automated.

### Western blotting

Western blotting was carried out as in previous studies (e.g. refs.^5,7,10^). Transfected HEK-293 cells were incubated with collagen or collagen-mimetic DDR-selective peptides in the presence or absence of anti-DDR1 mAbs, as indicated in the Figure legends. Cells were lysed in 1% Nonidet P-40, 150 mM NaCl, 50 mM Tris, pH 7.4, 1 mM EDTA, 1 mM phenylmethylsulfonyl fluoride, 50 µg/ml aprotinin, 1 mM sodium orthovanadate, and 5 mM NaF. Aliquots of the lysates were analysed by reducing SDS-PAGE on 7.5% polyacrylamide gels. The gels were blotted onto nitrocellulose membranes. Blots were first probed with phospho-specific Abs, followed by horseradish peroxidase conjugated goat anti-rabbit secondary Abs, then stripped in Antibody Stripping Solution (Alpha Diagnostic International, San Antonio, Texas) and re-probed with anti-DDR1 Abs followed by horseradish peroxidase conjugated goat anti-rabbit secondary Abs. Signal detection was performed using Enhanced Chemiluminescence Plus (Amersham Biosciences) on a Typhoon FLA 9500 Imager (GE Healthcare Biosciences). Densitometry analysis of protein band intensities was performed using ImageStudio^TM^ Lite (LI-COR Biosciences, UK).

### Flow cytometry

To determine binding of collagen, transfected COS-7 cells were removed from the plates with non-enzymatic cell dissociation solution (Sigma). Cells were incubated on ice with 20 μg/ml collagen I in 1% (w/v) BSA/PBS for one hour. Cells were then incubated on ice with mouse anti-DDR1 mAb 7A9 IgG1 and mouse anti-collagen I IgG2b, before fixing with 4% PFA/PBS for 15 minutes on ice. Secondary Abs were anti-mouse IgG1 Alexa Fluor-488 and anti-mouse IgG2b Alexa Fluor-555. The viability dye LIVE/DEAD Fixable Far Red (Molecular Probes) was used for gating on live cells. To determine compensation settings, single-staining of cells was done for each fluorophore. Background staining levels were determined by staining mock-transfected cells. Cells were analysed on a BD LSRFortessa flow cytometer (BD Biosciences) using BD FACSDiva software 6.0 (BD Biosciences) and further analysed on FlowJo software 10.2 (Tree Star, Inc.). The threshold for DDR1 staining was set to include 99% of the mock transfected cells. To quantify the average DDR1 expression level, the median values of the DDR1 stain was calculated for all cells above the threshold value. To determine collagen binding, the median value of the collagen stain was calculated for all cells expressing DDR1. To compare the collagen binding of cells expressing different DDR1 constructs, the collagen stain median values were weighted to the median DDR1 expression value.

To determine binding of DDR-selective peptide, transfected HEK-293 cells were dissociated as above and either singly incubated on ice with primary Ab (anti-DDR1 mAb 7A9) or biotinylated DDR-selective peptide, or were incubated with mAb 7A9 after pre-incubation with biotinylated DDR-selective peptide, diluted in 1% BSA/PBS. Following this, cells were incubated with anti-mouse IgG-FITC and/or Streptavidin Alexa Flour-546, as required, diluted in 1% BSA/PBS. Cells were fixed in 2% PFA/PBS before analysis on a BD LSRFortessa cell analyzer, as above. To compare peptide binding of cells expressing different DDR1 constructs, the peptide stain mean fluorescent intensity values were normalised to respective mean DDR1 stain values.

### Quantification of immunostain texture by the ‘zero-crossing method’

To assess the texture of the DDR1 surface immunostain in an unbiased manner, we developed automated cell image quantification suitable for uniform homogeneous textures like the ones observed in the DDR1 images. The method is implemented in Matlab (Mathworks) and available as the ‘CIDR’ program at https://github.com/yalexand/ALYtools. First, regions of interest (ROIs) are segmented in the image by using Gaussian gradient signatures at two different scales. Segmentation is designed to detect texture of interest while excluding image areas compromised by out of focus light or by bright membrane ruffles not related to the texture. Depending on cell morphology, segmentation results in sectioning each cell into 1–4 ROIs (or, representative texture patches). Then for texture quantification purposes, the original cell image is locally average-subtracted in order to exclude background variations due to uneven staining and illumination; a typical mask size for the local averaging is about 10 μm. The resulting reference image has an average intensity of zero, and the remaining intensity variations are only due to texture. This reference is used to quantify the ROIs (patches) by analysing the intensity profiles along random lines drawn across the ROI, which is done by counting the number of times the intensity profile crosses the zero level. The more times the intensity profile crosses the zero level, the finer the texture. The process is repeated several thousand (typically 2000) times for each patch in the image, and a mean number for each patch is calculated per unit length of the line. The outputted mean number is called the mean ZC (zero crossing linear density) score. The advantage of ZC score is that it is insensitive to a signal’s amplitude. A higher ZC-score for the patch indicates a finer texture, and conversely a lower ZC-score indicates a coarser texture.

### Statistical analysis

Experiments were performed at least three times, unless otherwise stated. Blots and images show representative examples. Statistical analysis was carried out using GraphPad Prism 7.00 for Windows (GraphPad software, LA Jolla, CA). The data were first tested for normality. Where appropriate, one-way or two-way ANOVAs were carried out, as indicated in the Figure legends, followed by the indicated post-hoc tests. Statistical significance was set at a p-values < 0.05.

## Supplementary information

Supplementary Information

## Data Availability

Materials and associated protocols from this manuscript are available upon request from the corresponding author.
