# Supplementary material for: DDR1 autophosphorylation is a result of aggregation into dense clusters
Source: Sci Rep. 2019 Nov 19;9:17104. doi: 10.1038/s41598-019-53176-4 (PMC6863838; doi:10.1038/s41598-019-53176-4)
Supplement: Supplementary file 1 — Supplementary Information [file 41598_2019_53176_MOESM1_ESM.pdf]

SUPPLEMENTARY FIGURES

TITLE: DDR1 autophosphorylation is a result of aggregation into dense clusters

Authors – David S Corcoran, Victoria Juskaite, Yuewei Xu, Frederik Görlitz, Yuriy Alexandrov, Christopher Dunsby, Paul MW French and Birgit Leitinger

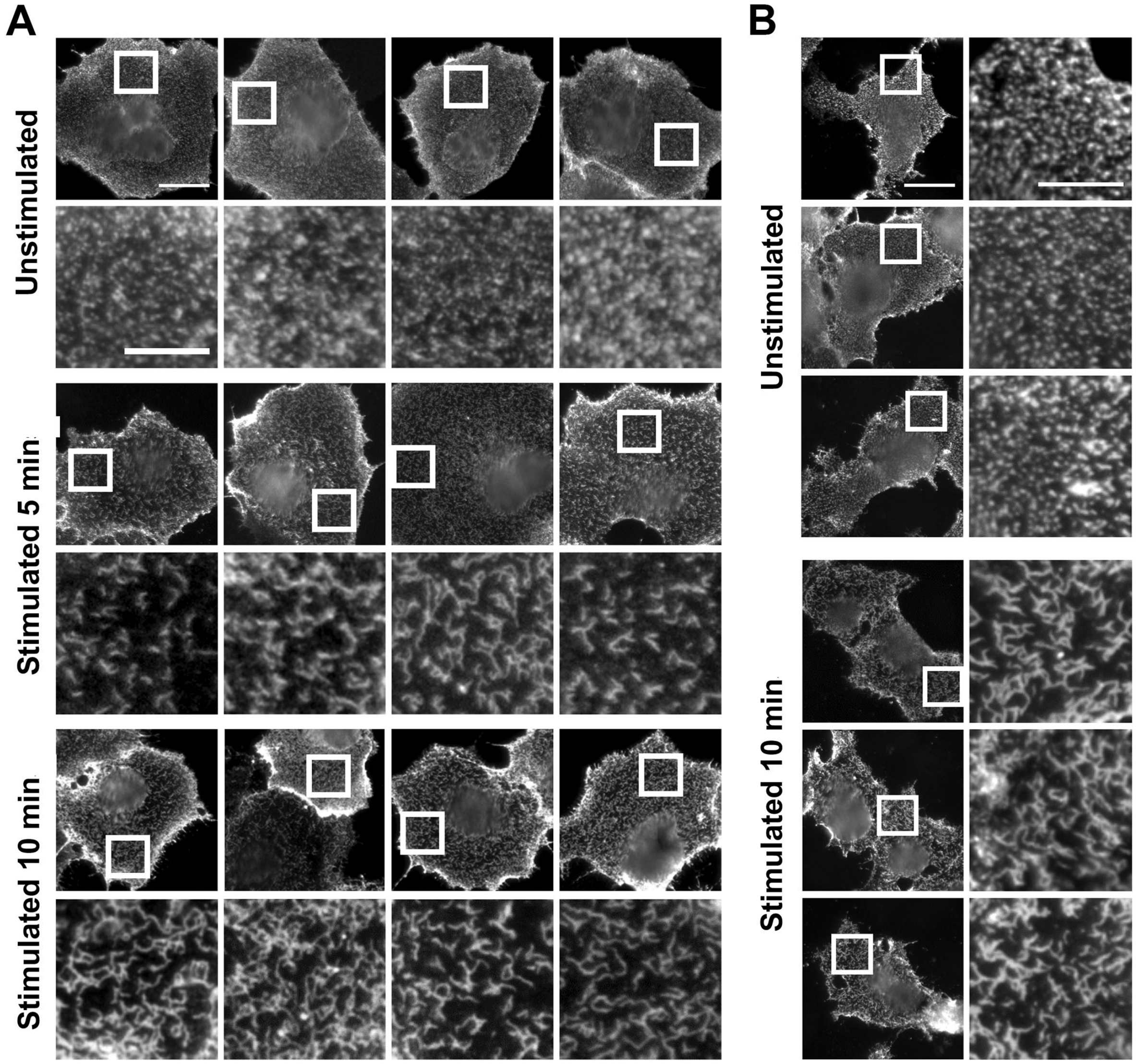

Figure S1

DDR1 redistributes on the cell surface with collagen stimulation. COS-7 cells transiently expressing DDR1 (A) or Flag-DDR1 (B) were stimulated with collagen I for 5 or 10 minutes at 37°C or left unstimulated, then incubated on ice with anti-DDR1 mAb 7A9 (A) or anti-Flag M2 mAb (B), before fixation and secondary Ab staining. Cells were imaged using a widefield microscope. White boxes indicate corresponding areas shown at higher magnification in lower rows (A) or in columns to the right (B). Scale bars, 30 µm (upper images in A or left images in B) or 10 µm (enlarged images). At least 30 cells were imaged for each condition.

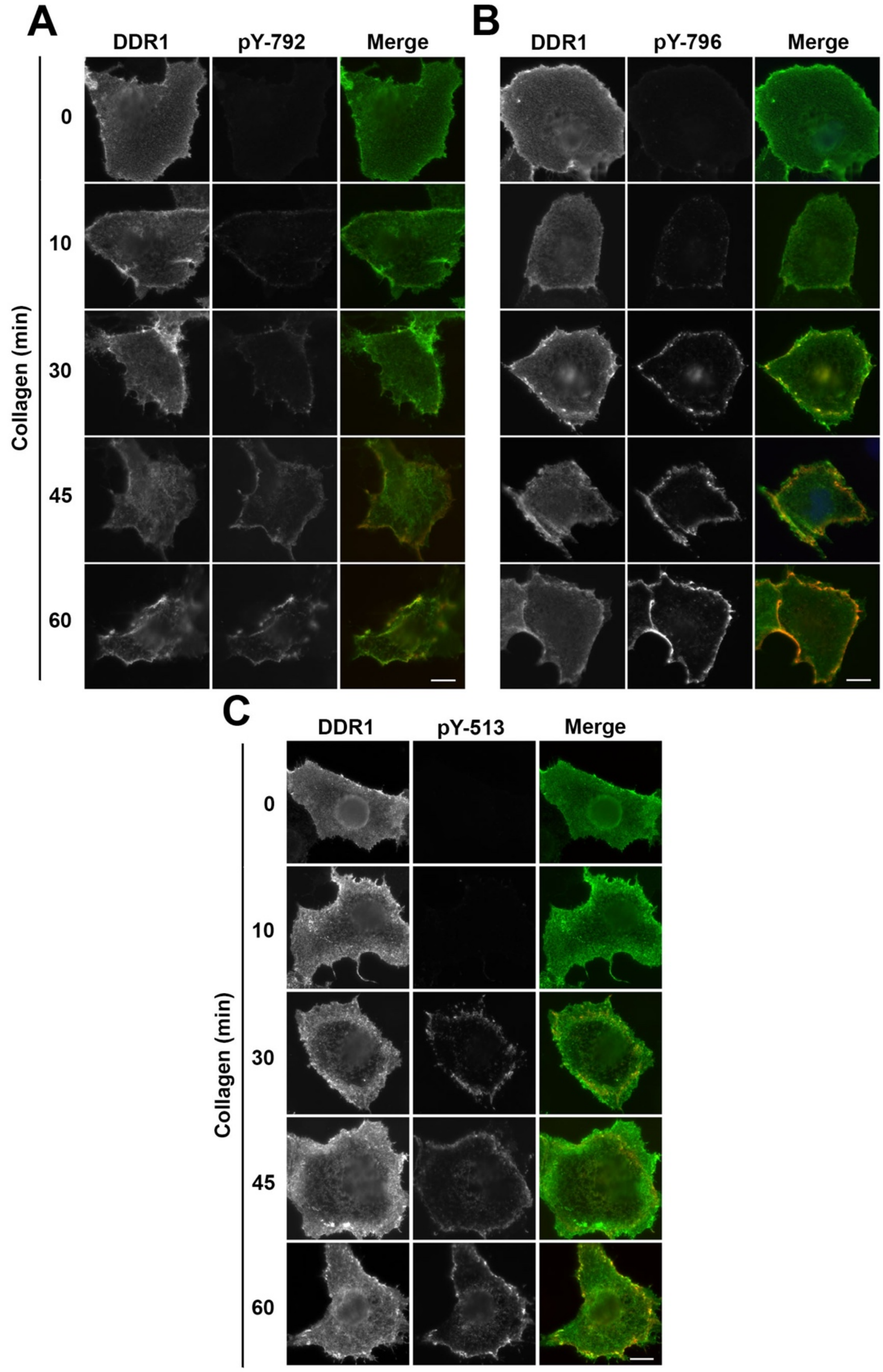

Figure S2
Collagen-induced DDR1 phosphorylation. COS-7 cells transiently expressing DDR1 were stimulated with collagen I for up to 60 minutes at 37°C or left unstimulated, then incubated on ice with anti-DDR1 mAb 7A9, before fixation, permeabilisation and immunostaining for phospho-tyrosine 792 (pY792) **(A),** phospho-tyrosine 796 (pY796) **(B)**, or phospho-tyrosine 513 (pY-513) **(C)**. Cells were imaged using a widefield microscope. Scale bars, 20 µm.

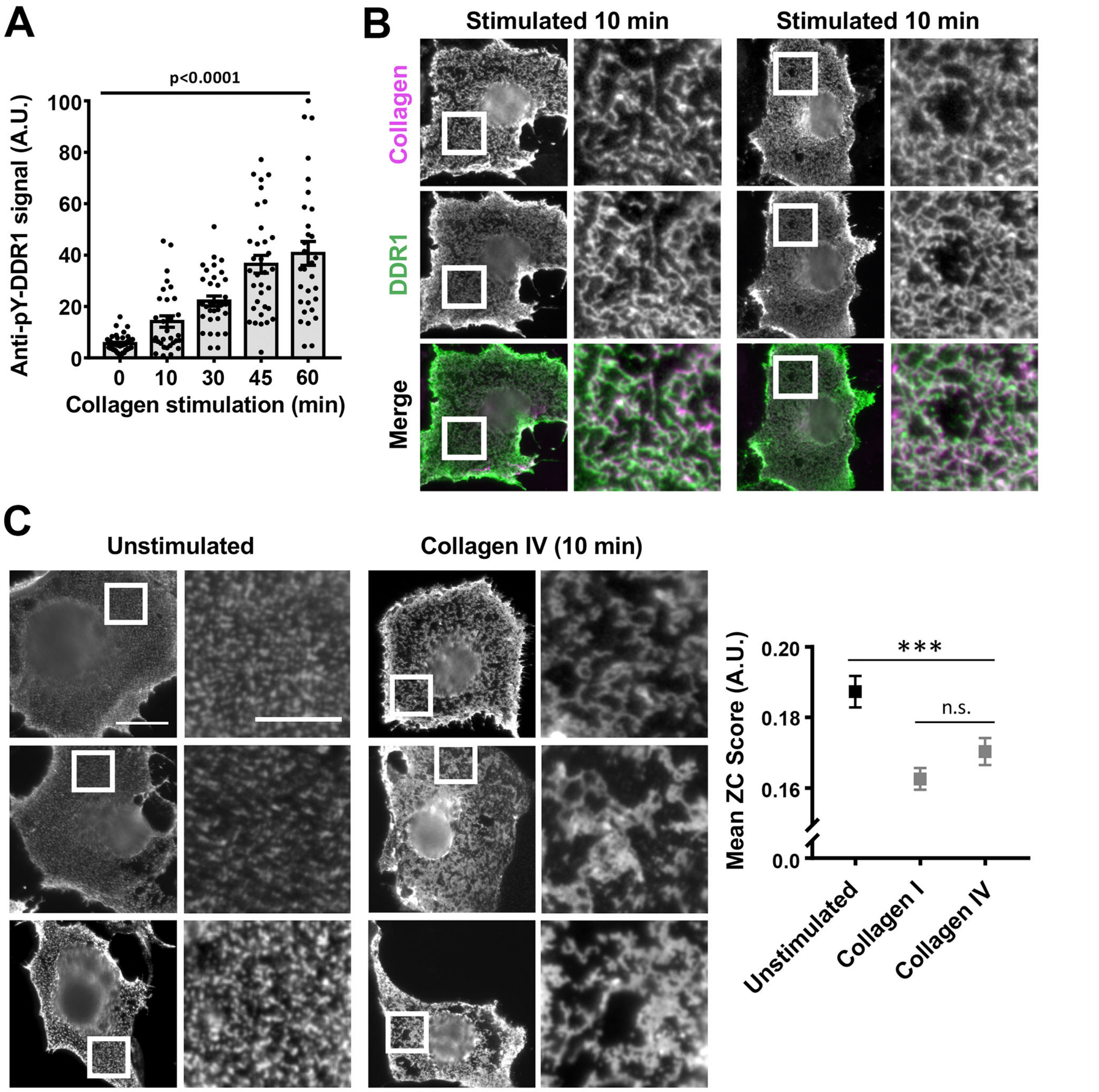

Figure S3

DDR1 distribution induced by collagen I and collagen IV. (A). Data from one of the experiments shown in Figure 2C. Each datapoint is the mean pY-DDR1 signal for one cell; bars show the mean value for each stimulation time, all datapoints were normalised so that the lowest and highest value were 0 and 100 A.U., respectively (n = 31-37). p<0.0001 (one-way ANOVA ($F(4, 163)=26.7$). **(B)** Further examples of data shown in Figure 2E. Green: DDR1 staining, magenta: collagen I staining. **(C)** COS-7 cells transiently expressing DDR1 were either stimulated with collagen IV for 10 minutes at 37°C or left unstimulated, then incubated on ice with anti-DDR1 mAb 7A9, before fixation, and secondary Ab staining. Cells were imaged using a widefield microscope. White boxes in left columns indicate corresponding areas shown at higher magnification in columns to the right. Scale bars, 30 µm (left image) or 10 µm (enlarged image). Graph shows mean ZC scores + SEM. ****p<0.0001 (one-way ANOVA ($F(2, 124) = 4.34$, p<0.0001). Post-hoc planned comparisons: unstimulated vs collagen I stimulated cells (p<0.001), unstimulated vs collagen IV stimulated cells (p=0.015), and collagen I vs IV stimulated cells (p=0.415). N = 30 -51.

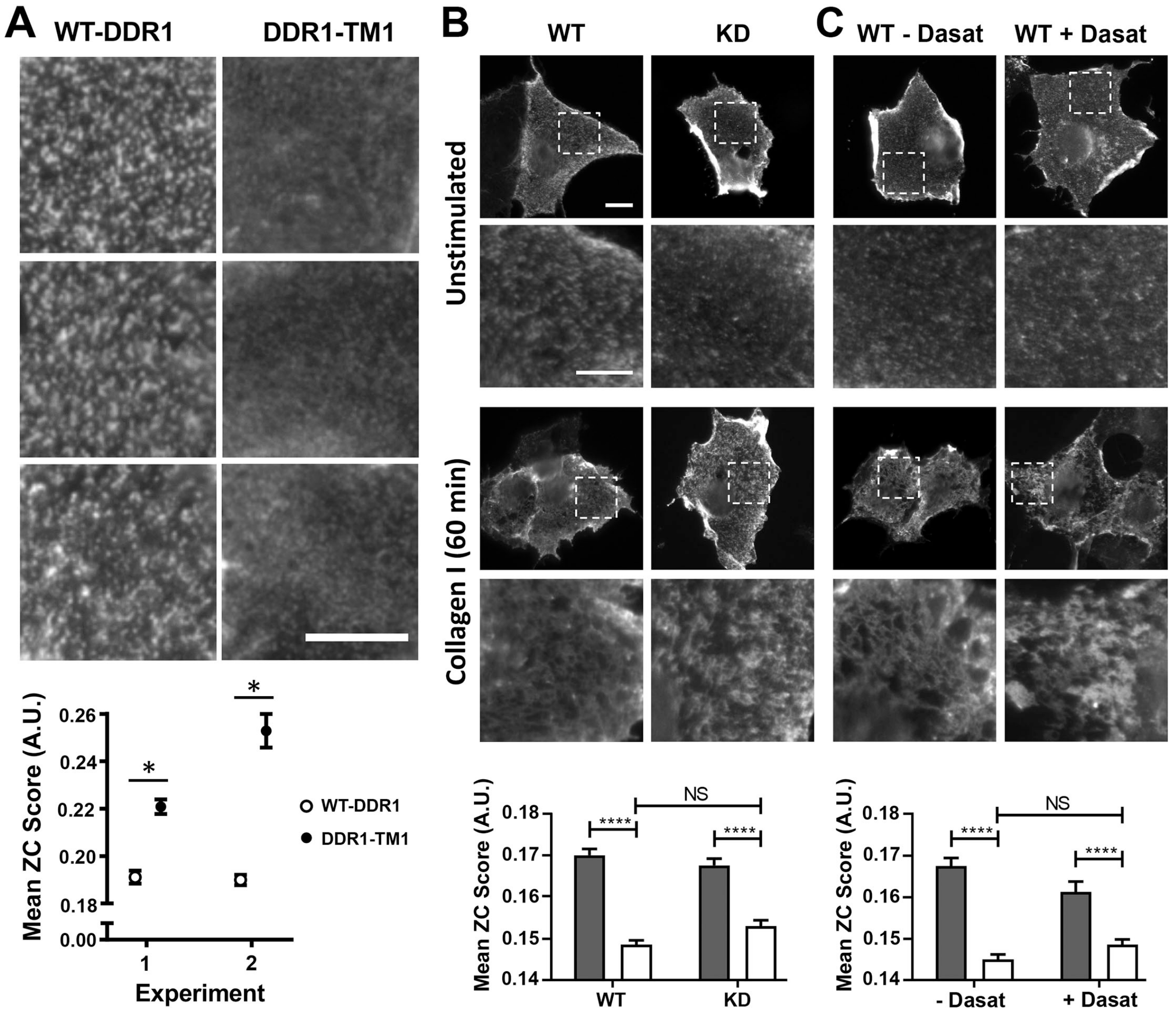

Figure S4

DDR1-TM1 has a finer surface immunostain compared to WT-DDR1, and collagen-induced DDR1 redistribution is independent of kinase activity. (A) COS-7 cells transiently expressing WT-DDR1 or DDR1-TM1 were incubated on ice with anti-DDR1 mAb 7A9, before fixation, and secondary Ab staining. Cells were imaged using a widefield microscope. Scale bar, 10 µm. The images show representative areas from 3 different cells. The graph shows mean ZC scores ± SEM from two independent experiments (experiment 1, n = 48 - 57; experiment 2, n = 52 -98). Two-way ANOVA interaction: $F(1,251) = 18.18$, $p<0.0001$. *$p<0.0001$, Tukey's multiple comparisons test. **(B and C)** COS-7 cells transiently expressing wild-type (WT) or kinase-inactive (KD) DDR1 were incubated with collagen I for 60 minutes at 37°C or left unstimulated, in the presence (WT + Dasat) or absence (WT - Dasat) of 10 nM dasatinib, as indicated. Cells were then incubated with anti-DDR1 mAb 7A9 on ice, before fixation and incubation with secondary Abs. Lower panels show magnified views of the boxed areas in the upper panels. Scale bar, 20 µm (upper panel) or 10 µm (enlarged image). Graphs show mean ZC score of surface DDR1 staining in DDR1-WT and DDR1-KD expressing cells stimulated with collagen I (white bars) or left unstimulated (grey bars), in the presence (+ Dasat) or absence (- Dasat) of dasatinib. Data show mean + SEM (n=200-300 regions from 30-45 cells from 2 independent experiments). NS, no significance; ****$p<0.0001$ (two-way ANOVA, followed by Bonferroni post hoc test).

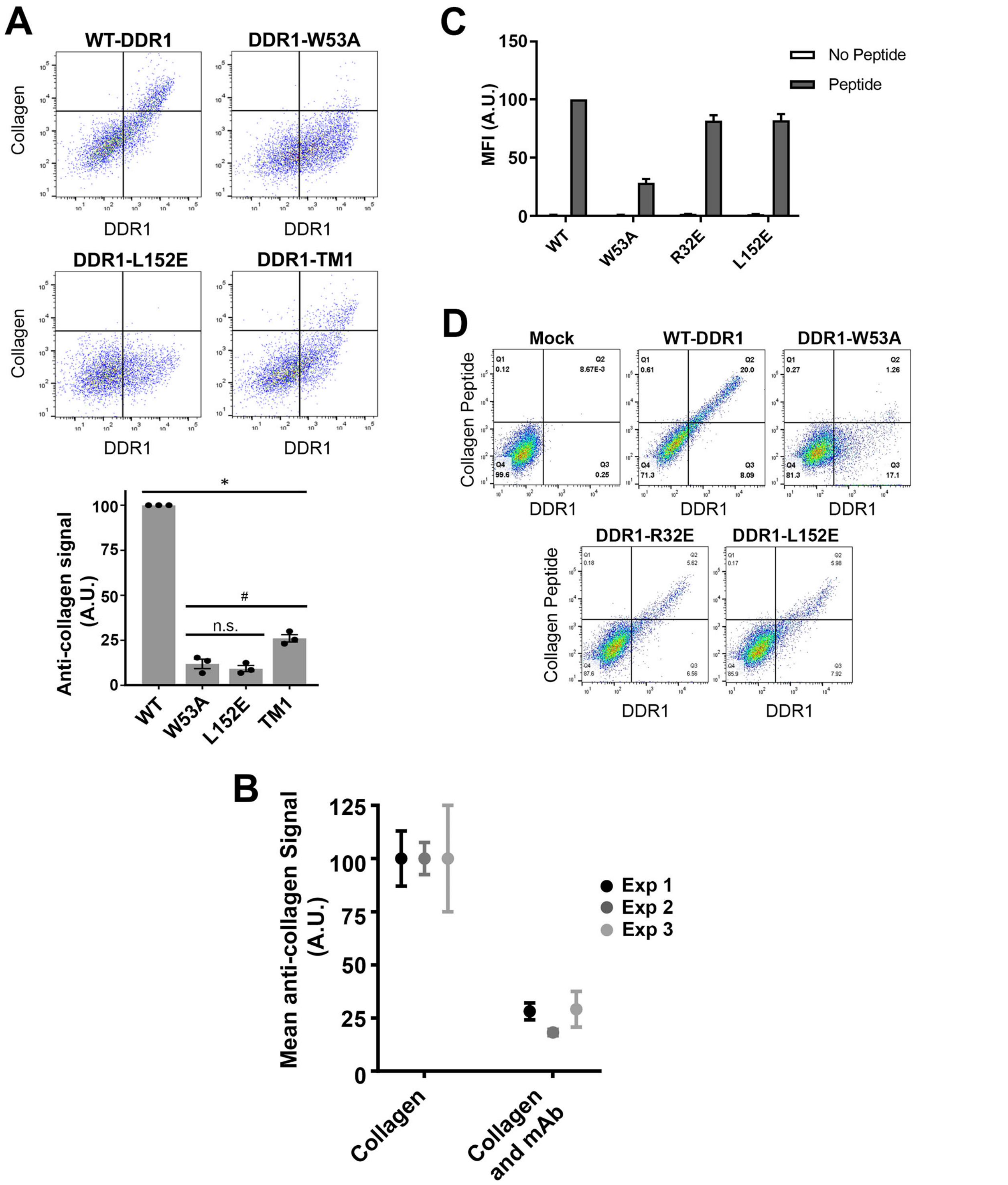

Figure S5

Flow cytometry to assess collagen binding to signalling defective DDR1 mutants. (A) COS-7 cells transiently expressing wild-type DDR1 or the indicated DDR1 mutant were incubated with collagen I for 60 minutes on ice. Cells were then incubated on ice with anti-DDR1 mAb 7A9 and anti-collagen-I lmAb, before fixation, and secondary Ab staining. Collagen binding and DDR1 expression was analysed by flow cytometry. The histograms show data from one experiment, the bar graph shows average data from three experiments, with each datapoint showing the median anti-collagen signal from an individual experiment and from only DDR1 expressing cells (defined as cells in the right-hand side quadrants in the histograms). Median values are weighted to the median DDR1 expression level for the construct and normalised so that the WT-DDR1 unstimulated and stimulated values were 0 and 100 A.U. respectively. Bars show the mean value + SEM (n=3 independent experiments). One-way ANOVA: F (4, 10) = 0.685, p<0.0001. Sidak's multiple comparisons test: WT-DDR1 vs all other constructs, *p<0.0001; DDR1-W53A vs DDR1-TM1, #p=0.00014; DDR1-W53A vs DDR1-L152E, n.s. For each

condition at least 10,000 cells were measured. **(B)** Data from 3 experiments as shown in Figure 3C, where cells were stimulated with collagen I for 60 minutes with or without mAb 7A9. The mean collagen-immunostain intensity was calculated for each condition, then normalised so that WT-DDR1 collagen-unstimulated and stimulated values were 0 and 100 A.U., respectively. Two-way ANOVA revealed a significant main effect of anti-DDR1 mAb incubation on anti-collagen signal ($F(1,152) = 73.38$, $p<0.0001$). At least 18 cells were imaged for each condition. **(C)** HEK-293 cells transiently expressing wild-type DDR1 or the indicated DDR1 mutant were incubated with biotinylated DDR selective collagen-mimetic peptide or anti-DDR1 mAb 7A9 for 60 minutes on ice, or left unstimulated. Cells were then incubated with Alexa Fluor-546 conjugated streptavidin or goat anti-mouse IgG-FITC. Peptide binding and DDR1 expression was analysed by flow cytometry. The bar graph shows the mean fluorescence intensity, normalised to respective DDR1 expression levels, from two independent experiments. The data were normalised so that the binding of peptide to wild-type DDR1 was set to 100 A.U. Mock transfected cells showed similar Alexa Fluor-546 signals as DDR1-W53A. The scatter plot shows data from a third independent experiment. 10,000 cells were measured for each condition

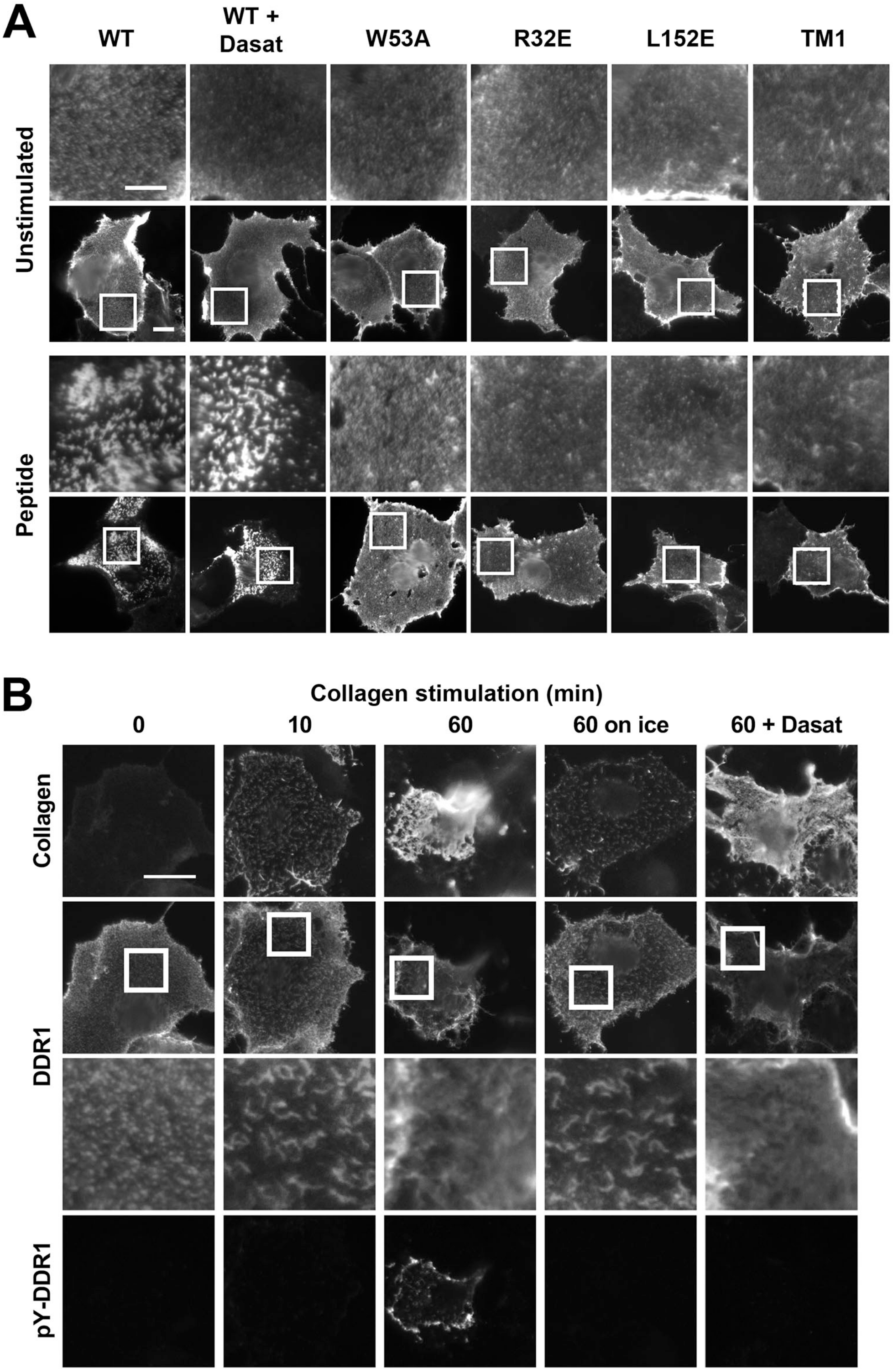

Figure S6

DDR-selective triple-helical peptide does not induce DDR1 redistribution of signalling defective DDR1 mutants, and collagen induced DDR1 redistribution in the presence of dasatinib. (A) COS-7 cells transiently expressing wild-type DDR1 or the indicated DDR1 mutant were incubated with collagen-mimetic triple-helical peptide for 60 minutes at 37°C or left unstimulated. Cells expressing wild-type DDR1 were incubated with peptide in the presence or absence of 10 nM dasatinib. Cells were then incubated with anti-DDR1 mAb7A9 Ab on ice, before fixation and incubation with secondary Abs. Top panels show magnified views of the boxed areas in the bottom panels. **(B)** COS-7 cells transiently expressing DDR1 were incubated with collagen I either at 37°C (for 0, 10, or 60 minutes), on ice (for 60 minutes), or in the presence of dasatinib (for 60 minutes at 37°C). Cells were then incubated on ice with anti-DDR1 mAb 7A9 and anti-collagen-I mAb, before fixation, permeabilisation and immunostaining for pY-DDR1. Lower panels for DDR1 show magnified views of the boxed areas in the top panels. All cells were imaged using a widefield microscope. Scale bars, 20 µm (left image in A), 10 µm (magnification in A) or 30 µm (B).

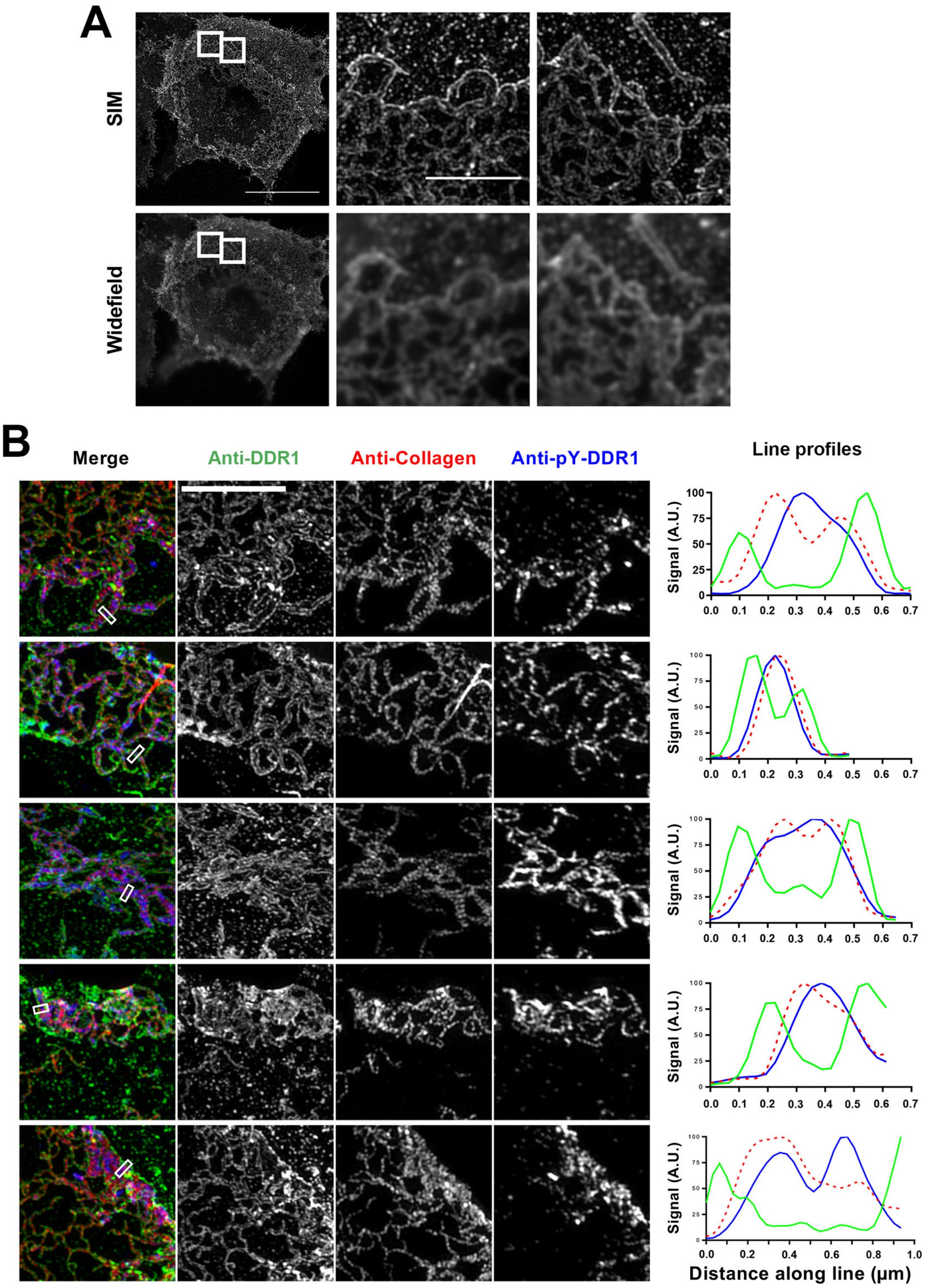

Figure S7

Anti-pY-DDR1 stain intensity correlated with presence of the anti-DDR1 double-walled structures. COS-7 cells transiently expressing DDR1 were stimulated with collagen I for 60 minutes at 37°C. **(A)** Cells were incubated on ice with anti-DDR1 mAb 5D5, before fixation, and secondary Ab staining. **(B)** Cells were incubated on ice with anti-DDR1 mAb 7A9 and anti-collagen-I lmAb for 60 minutes, before fixation, permeabilisation and immunostaining for pY-DDR1. 3D-SIM images were acquired using a Zeiss ELRYA microscope; images are from a maximum intensity projection of all 15 slices (A) or from one slice (B). White boxes indicate corresponding areas shown at higher magnification in columns to the right (A). Scale bars, 30 µm (left image in A), 3 µm (enlarged image in A) or 5 µm (B). Intensity of the three stains was measured across the lines shown in B (with a line width of 200 nm); the data were normalised so that the lowest and highest value from each stain was 0 and 100 A.U. At least 10 cells were imaged for each condition.

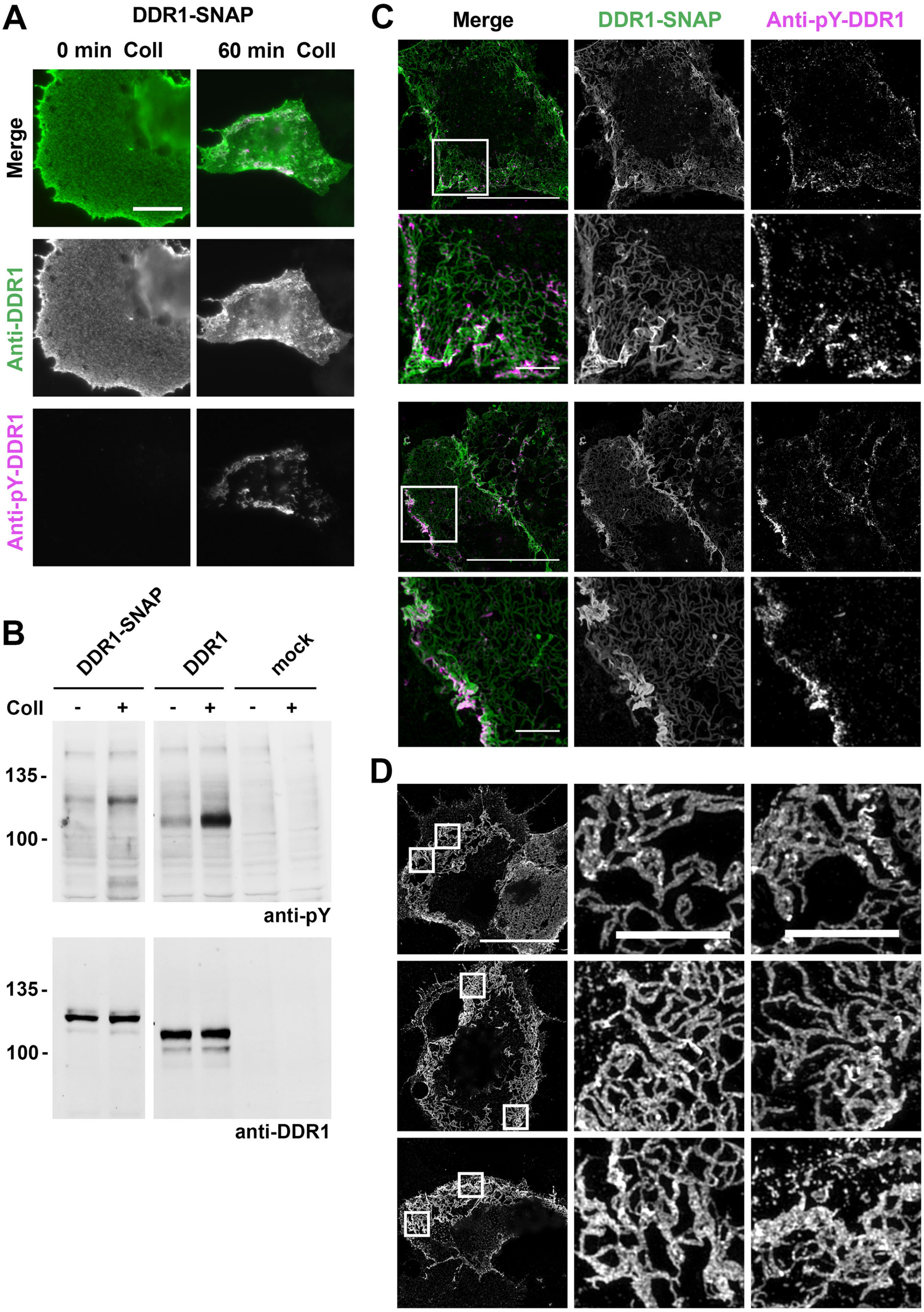

Figure S8

Collagen-induced phosphorylation and aggregation of DDR1-SNAP. (A) COS-7 cells transiently expressing DDR1-SNAP were stimulated with collagen I for 60 minutes at 37°C or left unstimulated, then incubated on ice with anti-DDR1 mAb 7A9, before fixation, permeabilisation and immunostaining for pY-DDR1. Cells were imaged using a widefield microscope. Scale bar,30 µm. At least 10 cells were imaged for each condition. **(B)** HEK-293 cells transiently expressing DDR1-SNAP1 or WT-DDR1 were stimulated with collagen I for 60 minutes at 37°C or left unstimulated. Cells were then lysed and analysed by SDS-PAGE and Western blotting. The blot was probed with anti-pY-513-DDR1 Ab and re-probed with anti-DDR1 (lower blot). The positions of molecular mass markers are indicated on the left in kDa. **(C)** COS-7 cells transiently expressing DDR1-

SNAP were incubated with SNAP-Surface Alexa Fluor-546 in DMEM for 60 minutes at 37°C, then stimulated with collagen I for 60 minutes at 37°C before fixation and immunostaining for pY-DDR1. **(D)** COS-7 cells transiently expressing FLAG-DDR1 were stimulated with collagen I for 60 minutes at 37°C, then incubated on ice with anti-FLAG mAb for 60 minutes, before fixation, and secondary Ab staining. **(C and D)** 3D-SIM images were acquired using a Zeiss ELRYA microscope. Images are from a maximum intensity projection of all 15 slices. White boxes indicate corresponding areas shown at higher magnification in lower images (C) or in right images (D). Scale bars, 20 µm (upper images in C), 3 µm (enlarged images in C), 30 µm (left image in D) or 5 µm (enlarged images in D). At least 10 cells were imaged for each condition.
